# Data‐Driven Feedback Identifies Focused Ultrasound Exposure Regimens for Improved Nanotheranostic Targeting of the Brain

**DOI:** 10.1002/advs.202517834

**Published:** 2026-01-07

**Authors:** Hohyun Lee, Victor Menezes, Shiqin Zeng, Chulyong Kim, Cynthia M. Baseman, Jae Hyun Kim, Samhita Padmanabhan, Pranav Premdas, Naima Djeddar, Anton Bryksin, Nikhil Pandey, Pavlos Anastasiadis, Anthony J. Kim, Tobey J. MacDonald, Chetan Bettegowda, Graeme F. Woodworth, Felix J. Herrmann, Costas Arvanitis

**Affiliations:** ^1^ Woodruff School of Mechanical Engineering Georgia Institute of Technology Atlanta Georgia United States; ^2^ School of Computational Science and Engineering Georgia Institute of Technology Atlanta Georgia United States; ^3^ School of Interactive Computing Georgia Institute of Technology Atlanta Georgia United States; ^4^ Coulter Department of Biomedical Engineering Georgia Institute of Technology and Emory University Atlanta Georgia United States; ^5^ School of Electrical and Computer Engineering Georgia Institute of Technology Atlanta Georgia United States; ^6^ Institute of Bioengineering and Bioscience Georgia Institute of Technology Atlanta Georgia United States; ^7^ Department of Neurosurgery University of Maryland School of Medicine Baltimore Maryland United States; ^8^ Brain Tumor Program Marlene and Stewart Greenebaum Comprehensive Cancer Center University of Maryland Baltimore Maryland United States; ^9^ Department of Diagnostic Radiology and Nuclear Medicine University of Maryland School of Medicine Baltimore Maryland United States; ^10^ Department of Bioengineering University of Maryland Baltimore Maryland United States; ^11^ Aflac Cancer & Blood Disorders Center Department of Pediatrics Emory University School of Medicine Atlanta Georgia United States; ^12^ Department of Neurosurgery John Hopkins University School of Medicine Baltimore Maryland United States; ^13^ School of Earth and Atmospheric Sciences Georgia Institute of Technology Atlanta Georgia United States

**Keywords:** blood‐brain barrier, brain cancer, focused ultrasound, liquid biopsy, machine learning, nanoparticle, theranostics

## Abstract

The blood‐brain barrier (BBB) renders the delivery of nanomedicine in the brain ineffective and the detection of circulating disease‐related DNA from the brain unreliable. Here, we demonstrate that microbubble‐enhanced focused ultrasound (MB‐FUS) mediated BBB opening, supported by large‐data models predict sonication regimens for safe and effective BBB opening. Importantly, a closed‐loop MB‐FUS controller augmented by machine learning (ML‐CL) expands the treatment window, as compared to conventional controllers, by persistently and proactively maximizing the BBB permeability while preventing tissue damage. By successfully scaling up from mice to rats and from healthy to diseased brains (glioma), ML‐CL rendered the BBB permeable to large nanoparticles and markedly improved the release and detection of reporter gene DNA from tumors in blood. Together, our findings reveal the potential of data‐driven feedback to support the development of next‐generation AI‐powered ultrasound systems for safe, robust, and efficient nanotheranostic targeting and treatment of brain diseases.

## Graphical abstract

Machine learning models predict in real time the onset of harmful microbubble collapse during microbubble‐enhanced focused ultrasound (MB‐FUS) and enable dynamic adjustment of sonication to prevent cavitation‐induced damage. This predictive control expands the safe operating window for bloodbrain barrier opening, enhancing nanoparticle delivery and tumor biomarker detection in blood.

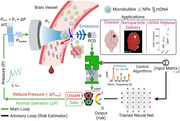

## 1 Introduction

The blood‐brain barrier (BBB) constitutes a highly selective semi‐permeable multi‐cellular interface between the vascular lumen of capillaries and the brain parenchyma that tightly regulates the transport of macromolecules to support the metabolic demands of the brain and the clearance of soluble waste products [1, 2, 3, 4]. Due to its properties, the BBB hinders the effective delivery of a wide range of therapeutic agents (larger than 400 Da [5, 6]) into the brain, including highly potent nanotherapeutics [6, 7, 8, 9]. It also limits the reliable release of soluble biomolecules into circulation – molecules that are associated with disease formation, progression, and responses to therapy – and are therefore essential for minimally invasive diagnosis and longitudinal monitoring of brain diseases through liquid biopsy techniques [10, 11]. Hence, strategies to safely and effectively overcome the BBB – including its partially compromised, yet highly heterogeneous permeability during disease progression [6, 12] – are critical for the diagnosis, treatment, and monitoring of central nervous system (CNS) diseases.

Circulating microbubbles (MB) upon focused ultrasound (FUS) exposure (sonication) can transiently increase the BBB permeability in a disease‐agnostic way [13, 14, 15] and facilitate mass transport across this biological interface in an exposure‐dependent manner [16, 17]. Notably, MB‐FUS has been shown to increase the effective delivery of various therapeutic agents – including a broad spectrum of nano‐formulations [16] – across a wide range of brain diseases [16, 18, 19]. Despite promising preclinical findings, the quest to deliver an increasing fraction of intravenously administered nano‐carriers into the brain [20] combined with the need to incorporate larger and more potent cargos (e.g., gene editing nano‐systems) that inevitably require larger constructs (≥60 nm) [21], is probing scientists and clinicians to employ FUS exposures close to safety limits to maximize their penetration across the BBB [22, 23, 24, 25, 26, 27]. Similar advancements and challenges have also been observed in the use of MB‐FUS to promote the release of disease‐associated soluble molecules from the brain into circulation. The integration of MB‐FUS with liquid biopsy techniques, whereby biomarkers from sonicated tissues are transiently enriched in the circulation, enables the development of novel assays for diagnosing and characterizing brain diseases [28, 29, 30, 31, 32, 33, 34, 35]. This includes the release of larger and scarcer molecules, such as circulating‐tumor DNA (ctDNA, fragments ≥ 60 kDa), which are critical for improving the sensitivity, specificity, and ultimately the diagnostic utility of these assays. However, such sono‐interrogation approaches must carefully balance the need for aggressive sonication to improve biomarker release against the risk of inducing brain injury [29, 30, 31, 32, 35].

Balancing BBB opening efficacy with safety requires effective and real‐time control of the cerebrovascular MB dynamics [16], a highly nonlinear phenomenon that is prone to instabilities, such as MB collapse (i.e., inertial cavitation) that has long been linked to tissue damage [36]. Recent investigations have shown that recording and analyzing the acoustic emissions (AE) generated by MBs’ nanoscale oscillations, which incorporate strong nonlinear acoustic components, provides a robust way to adjust the sonication settings [16, 37]. The sonication amplitude can be modified in real‐time to attain the prescribed strength of nonlinear acoustic components (i.e., Harmonics and/or Ultra‐Harmonics) that are good proxies to stable oscillations and positively correlate with safe BBB opening. While this principle has formed the basis for developing real‐time closed‐loop methods to maximize BBB opening [17, 38, 39, 40, 41, 42], safety remains a concern as all current control methods are reactive to the onset of broadband emission: a warning sign associated with MB collapse. That is, they rely on the detection of broadband emission events before triggering a “safety response” (i.e., rapid pressure drop). As a result, small damage from repeated treatment sessions targeting neurologically functional brain regions (e.g., tumor invading healthy brain or neurological diseases like Alzheimer's) can be compounded [43, 44, 45, 46]. Consequently, this leads to conservative exposure settings during MB‐FUS and a very narrow acoustic treatment window (∼50 kPa) [16, 36]. The latter not only complicates the effective clinical translation and broad dissemination (i.e., similar to US imaging) of this technology, but also challenges its ability to consistently attain clinically meaningful endpoints related to the diagnosis, treatment, and treatment monitoring of CNS diseases.

Data‐driven methods, such as machine learning (ML), are powerful tools for identifying nonlinear relationships in complex datasets. While ML has been widely applied to ultrasound imaging and diagnostic tasks [47], its use in therapeutic ultrasound, particularly for microbubble dynamics and their control, remains limited. Here, we employ data‐driven models to extract precursor signals (i.e., patterns) [48, 49] that are within the MB AE to study and analyze the transition from stable to inertial MB oscillation. Through comprehensive analysis and training of the models, using more than 54 000 AE datasets collected in MB‐FUS experiments in mice, we discover multi‐dimensional relationships in features derived from nonlinear MB AE that can predict the onset of broadband emissions with high sensitivity. By integrating the trained model into a real‐time closed‐loop MB AE feedback controller, we show that it can augment the MB‐FUS acoustic treatment window by persistently and proactively maximizing the BBB permeability while preventing tissue damage. We show that this data‐driven feedback overcomes barriers to the delivery of nanoparticles and markedly improves the release of soluble biomarkers (protein and ctDNA) in orthotopic glioma tumor model in rats. Collectively, our findings demonstrate that, by expanding the treatment window, data‐driven feedback can augment ultrasound nanotheranostic targeting of brain tumors and support the development of next‐generation AI‐powered ultrasound systems for improved diagnosis, treatment, and monitoring of brain diseases.

## 2 Results

### 2.1 Multilayer Perceptron Achieves High Sensitivity in Predicting Broadband Emissions

**FIGURE 1:**
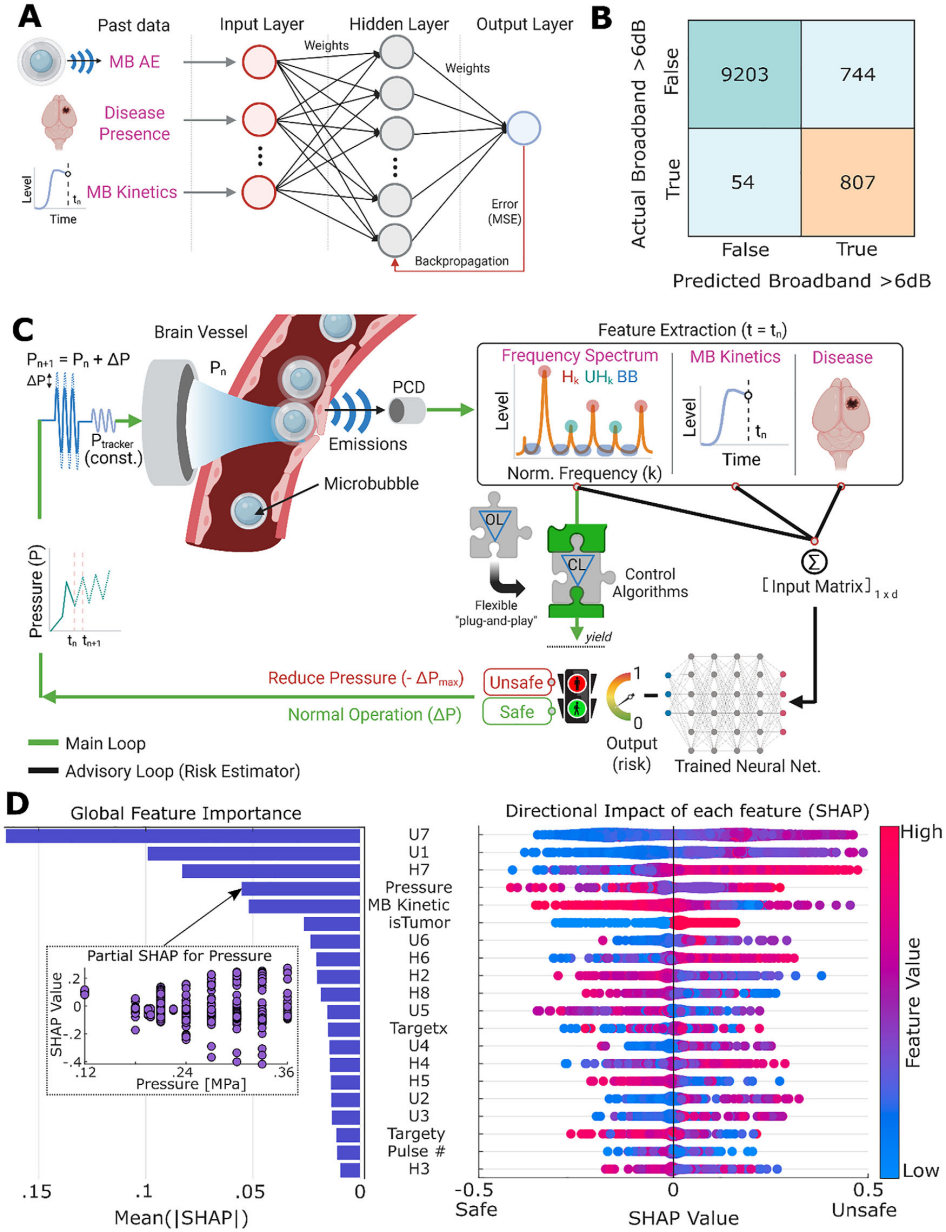
Multilayer perceptron model training, interpretation, and its integration with the controller. (A) Schematic of simple proof‐of‐concept MLP model with one hidden layer with 10 neurons. Input features are described in Table 1. (B) Confusion matrix for MLP model testing; Numbers in the matrix indicate the number of data points, with the top left, top right, bottom left, and bottom right being true negative, false positive, false negative, and true positive, respectively. (C) Schematic of ML‐assisted closed‐loop MB‐FUS controller. MLP model's output is used as an additional safety layer (advisory loop) to override closed‐loop controller's decisions upon positive prediction. This flexible design allows its universal compatibility with various control algorithms. (D) SHAP value analysis onto MLP model. All training datasets were used for analysis without random sampling. The left shows global importance calculated from the absolute value of SHAP values. Partial SHAP analysis of a single feature, pressure, shows a weakly positive correlation between pressure and SHAP, but with high variance, which indicates the effect of pressure onto model is not monotonic. For the right (SHAP analysis), being closer to red indicates higher values of each feature. SHAP value (*x*‐axis) represents the contribution of each feature's value to the output of the model (positive SHAP value indicates the tendency of positive broadband prediction by the model).

In this proof‐of‐concept investigation, we applied a supervised classification machine learning (ML) model trained on 54 040 acoustic emission (AE) datasets from BBB opening experiments across 415 distinct brain targets in 114 mice. All training data and experimental results presented in this study were acquired using a custom‐built US‐guided FUS system (USgFUS) operating at 0.5 MHz excitation frequency (see Methods) [17]. As a proof‐of‐concept ML model, we employed a neural network model known as multilayer perceptron (MLP) consisting of fully connected neurons with one hidden layer (10 neurons) and a sigmoid activation function (Figure 1A). Despite being a simple model, this structure allows the model to learn complex relationships between input dataset—MB AE, for instance—and broadband emission that are difficult to capture with conventional statistical tools [50]. Moreover, when prior knowledge about feature relationships (i.e., MB AE vs. broadband emission) is limited or unavailable, MLPs—being a universal function approximators—offer a flexible solution compared to other ML models. We then trained the MLP model using Levenberg–Marquardt algorithm [51]. The inputs to the model included 12 features: primarily MB AE in the ultra‐harmonic frequency range, which are considered to be closely related to inertial cavitation and brain damage [39], as well as additional variables such as MB kinetics and the presence of disease (cancer) in the targeted tissue (see Table 1 for a complete list of features). Each dataset was labeled (i.e., ground truth labeling) based on whether a broadband emission event (defined as 6 dB above baseline noise) occurred in the subsequent sonication. We chose a 6 dB threshold because it corresponds to approximately three times the standard deviation of the baseline noise, which is approximately 2 dB across our data. Using three times the standard deviation provides a statistically conservative criterion for classifying broadband emission events that are highly unlikely to arise from noise fluctuations alone (*p* < 0.01), and this ensures that only clearly resolved cavitation‐related transient broadband events are positively labeled.

**TABLE 1:** Features and label for MLP model training.

Feature (*t* = T)					Label (*t* = T+1)
1‐7th Ultra‐harmonic	Pressure	Target Location	MB Kinetics	Tumor Presence	Broadband emission >6dB

Due to the inherent class imbalance (only 8% of total AE data contained broadband emissions) and to prevent the model from being biased toward the more frequent no‐broadband events, we performed under‐sampling [52] of the majority class (i.e., no‐broadband) (Figures S1 and S2). We then trained the MLP model using 80% of the under‐sampled data and tested it on the remaining 20% (see Methods). In our testing dataset, we found 93% (10 010/10 808) overall accuracy, 94% (807/861) sensitivity (true positive rate), and 52% (807/1551) precision (false positive vs. true positive), and 99% (9203/9257) specificity (true negative rate) in predicting broadband emission events (Figure 1B). Crucially, the retained high sensitivity suggests that the model remains highly effective at detecting true broadband emission events. While we acknowledge the relatively low precision (false positives), this reflects the model's bias toward conservativeness—favoring occasional false positive predictions over missed detections. In the context of clinical translation, considering broadband emissions’ scarcity and potential safety implications, this conservative behavior is one of the desirable traits especially when minimizing the risk of missing predictions is a priority. The high sensitivity further assures that the model is not simply over‐predicting indiscriminately, but rather making selective and reliable detections that align with our goal of safety‐focused clinical translation. We also confirmed that other machine learning models, such as support vector machine (SVM), had comparable performance (Figures S1 and S2). Importantly, we found that when it is necessary to improve the performance of the model (i.e., precision), more advanced deep learning techniques such as the attentive multilayer perceptron (AMP) model are highly potent (Figures S3 and S4, see ExperMethods and Supporting Information for detailed design). Together, these findings highlight the potential of ML methods to identify precursor patterns of future MB collapse. By anticipating rather than reacting to these adverse events, such data‐driven methods offer the potential for real‐time safety monitoring during MB‐FUS treatment (i.e., responding before MB collapse and associated brain damage has taken place).

### 2.2 Design and Interpretation of Machine Learning‐Assisted Closed‐Loop Controller (ML‐CL)

#### 2.2.1 Design of Machine Learning‐Assisted Closed‐Loop Controller Framework

Having established the predictive potential of the MLP model, we then designed a machine learning‐assisted closed‐loop MB‐FUS controller (ML‐CL) by integrating the trained MLP model into a closed‐loop controller (CL) algorithm we have recently proposed and validated [17]. CL algorithms operate by targeting a desired AE level (i.e., target level) and dynamically adjusting their input pressure based on real‐time feedback from the current AE level, with the goal of minimizing the error between the current and target AE levels [16]. In this framework, on top of the CL algorithm, we implemented the MLP as an additional safety layer (Figure 1C). This design preserves the controller's responsiveness to broadband emission events (i.e., reactive element) while introducing an additional predictive element that enables the controller to also anticipate broadband emissions and respond as if they have already occurred. Importantly, integrating MLP as an additional safety layer does not interfere with the controller's actions (i.e., controlling harmonic emissions) during normal operation but ensures that the MLP acts when necessary—overriding the controller's decision upon prediction of a broadband event during sonication. Perhaps the most salient feature of this design is its flexibility, as it allows the MLP to be integrated into any controller or system (provided there is sufficient AE data for training from ongoing trials or experiments) without altering its core functionality or features. Likewise, different ML models (see Supporting Information) can be integrated into this framework.

#### 2.2.2 Interpreting Machine Learning Models Offer a Data‐Driven Approach for Studying and Analyzing the MB Dynamics in Brain Vessels

In light of the above findings, we took this analysis one step further by attempting to interpret the trained MLP model and support a data‐driven analysis of MB dynamics. To interpret our MLP model, we assessed our results using Shapley Additive Explanations (SHAP) (Figure 1D). SHAP assigns each input feature a contribution score (SHAP Values), indicating how much it influences the model's output [53] and hence enabling quantification of their importance. While the original MLP model was trained using ultra‐harmonics as the MB AE features, we included both ultra‐harmonics and harmonics in the SHAP analysis to evaluate the broader importance of MB AE components (see Table 2 for a complete list of features). As indicated by SHAP values, we found that the top 7 contributing input features were strongly associated with the key physical characteristics of the MB dynamics and the configuration of the USgFUS system (Figure 1D). These were: (i) the passive cavitation detector's (PCD) peak sensitivity at 3.5 MHz, represented by 6th, 7th ultra‐harmonic and 7th harmonic; (ii) the MB dynamics strength and proximity to MB instabilities (i.e., bubble collapse), represented by 1st ultra‐harmonic, which are unique signatures of MB dynamics and have been used in the past to control the MB dynamics and establish safety thresholds [39, 54]; (iii) the number of MBs in focal region, represented by MB kinetics; and (iv) the peak negative pressure, which is a key driver of MB dynamics [55]. Interestingly, while lower‐order harmonics are often considered more useful in practice—owing to their efficient emissions [56] and effective transmission through the skull—our results suggest that the harmonics close to the receiver sensitivity (even up to 7th harmonic) exhibit similar significance. This highlights the importance of the system's design and tuning for recording MB AE. In our preclinical system, harmonics near 3.5 MHz (7th harmonic) were shown as critical, which suggests that controlling 7th harmonic (3.5 MHz) should be prioritized with this system configuration. Importantly, we incorporated this insight by designing the closed‐loop controller to regulate the 7th harmonic but preserving harmonic‐independent MLP model (see Section 2.1 and Table 1). That is, closed‐loop controller controls the 7th harmonic separately, while the MLP model (trained without harmonics) continues to predict precursors of broadband emission events. This structure allows the predictive model to be relatively insensitive to nonlinear artifacts (nonlinear propagation or system nonlinearity), although the controller still leverages the information provided by 7th harmonic, which is also the strongest signal available in our current system. We anticipate that this system‐specific result will be different for clinical systems, however our framework can be applied as is, using data from such systems. Our analysis also revealed that the presence of tumors can elicit a higher probability of broadband emissions, which is surprising, as this MB behavior has not been reported in the past despite extended preclinical and clinical investigations.

**TABLE 2:** Features and label for MLP SHAP analysis.

Feature (*t* = T)						Label (*t* = T+1)
2–8th Harmonic	1–7th Ultra‐harmonic	Pressure	Target Location	MB Kinetics	Tumor Presence	Broadband emission >6 dB

While most features showed consistent behavior—with high (red) or low (blue) feature values pushing the model's predictions in a clear direction, Figure 1D –SHAP analysis revealed a more complex pattern for sonication pressure. We found that while pressure was a highly influential variable, its effect on the model's output was not monotonic. This suggests that although sonication pressure plays a key role in shaping MB dynamics, its relationship with broadband emission is likely situation‐dependent. That is, despite a weak positive overall correlation (Figure 1D) between sonication pressure and broadband emission, this effect can be influenced by the skull and (transient) MBs’ concentration‐dependent dynamics, among others [57, 58]. Moreover, by analyzing our training dataset, we found a close correlation between MB kinetics (5th feature of importance according to MLP's SHAP values) and the strength of broadband emission, which suggested that stronger broadband emission likely occurs at higher (>70%) MB (transient) concentrations in the body after a bolus administration (Figure S5). This indicates that the temporal MB dose fluctuation, in addition to the total MB dose [58, 59, 60, 61], is an important safety consideration. Together with SHAP analysis, these findings underscore the inherent risk associated with the use of constant pressure sonications (currently widely used in clinics) and further support the use of “ramping up” (closed‐loop) control algorithms [17]. In aggregate, our results demonstrated the capability of ML models and data‐driven analysis of MB dynamics to predict instabilities in their behavior (i.e., MB collapse) and uncover subtle but potentially important features that shape their highly nonlinear response in vivo.

### 2.3 Safety and Efficacy Evaluation of Machine Learning‐assisted Closed‐Loop FUS Controller (ML‐CL) in BBB Opening

#### 2.3.1 Evaluation of ML‐CL at Low Exposure Condition (32 dB)

To evaluate the operation of the ML‐assisted closed‐loop controller (ML‐CL), we first performed MB‐FUS‐mediated BBB opening in healthy mice by targeting three regions in each brain hemisphere using the USgFUS system that we used to collect the training data. We selected a target level of 32 dB at 7th harmonic emission using the cavitation threshold curve identified from our training dataset (Figure S6A). We chose this target level because it was at the highest level within the linear (before plateauing) regime of the curve. With 32 dB target level, we found that the average and maximum pressure decisions (*P*_Avg_ and *P*_Max_, reported as peak negative pressures) of ML‐CL were 0.17 and 0.23 MPa, respectively (Figure S6B). Along with the pressure corresponding to 32 dB in the cavitation threshold model (*P*_Model_ = 0.14 MPa, Figure S6A), these pressures provided the necessary exposure conditions for implementing constant pressure open‐loop controllers (OL) to compare and benchmark the ML‐CL controller's performance. Unfortunately, the selected target levels and exposure settings were characterized by a low incidence of broadband emission events (3 events per 1800 pulses) and, as such, they did not allow us to fully expose their potential benefits of ML‐CL and tradeoffs between safety and efficacy (see below and Figure S6). Despite their shortcomings, they allowed us to establish feasibility for the proposed methodology in predicting and preventing scarce broadband emission events (See Supporting Information under Methods section and Figure S6). They also provided an upper safety limit for the conventional (i.e. reactive) control methods as well as a lower BBB opening limit (*P* ^32dB^_Avg_).

#### 2.3.2 Evaluation of ML‐CL at High Exposure Condition (36 dB)

**FIGURE 2:**
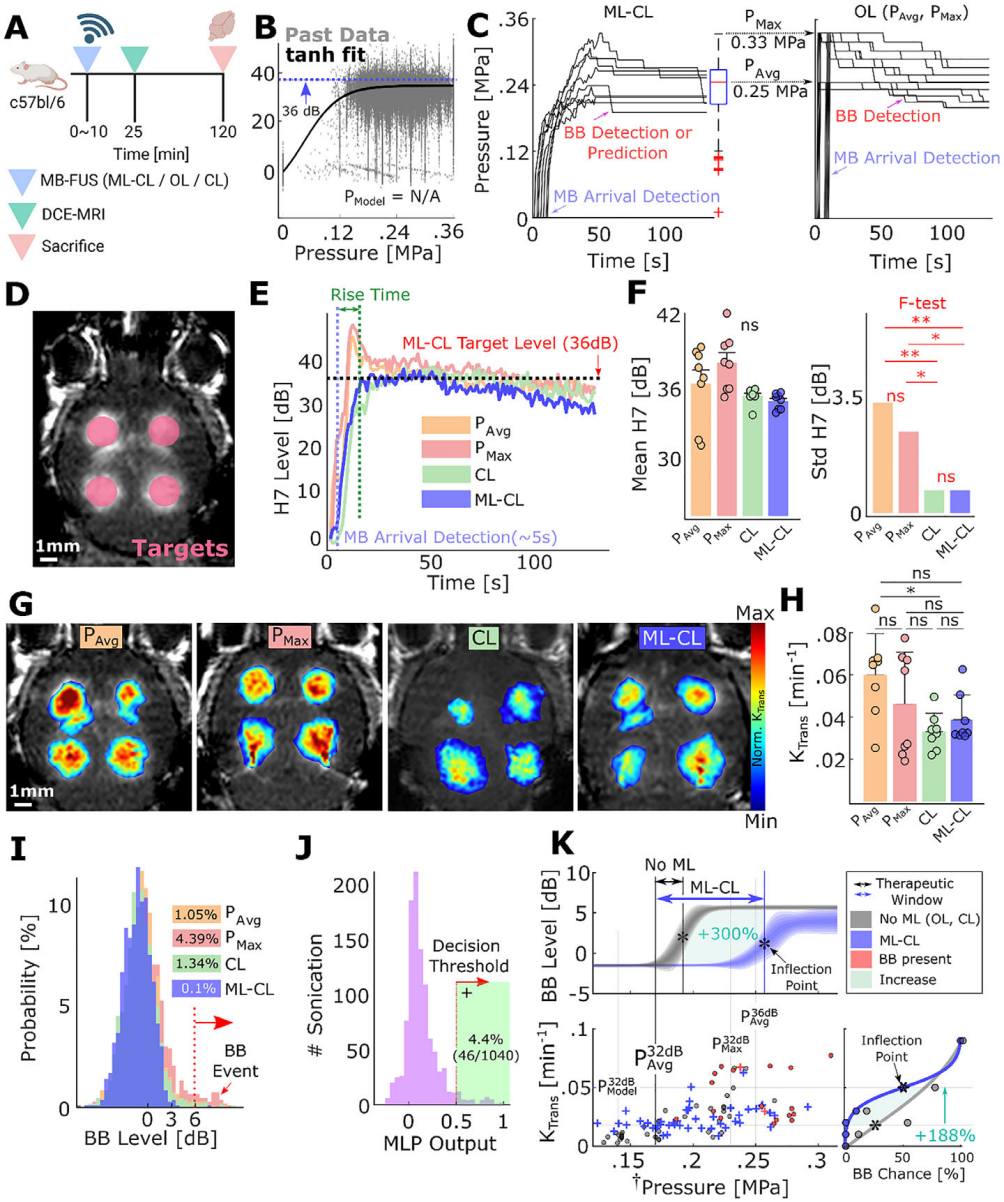
Treatment window expansion by ML‐CL. (A) Experimental workflow for all MB‐FUS controllers. (B) Cavitation threshold model from the training dataset, showing 7th harmonic AE vs. pressure; 36 dB target level was chosen above the model. (C) ML‐CL pressure decisions (left) and corresponding *P*_Max_ and *P*_Avg_ used for OL controllers (right); OL's pressure reduction indicates response to broadband emission events (> 6 dB). D) Representative MRI image for 36 dB target level sonication targets. Total 4 targets per mouse. (E) 7th harmonic emissions during sonication for each controller. *P*_Avg_:36.2 ± 3.3 dB, P_Max_ 37.9 ± 2.4 dB, CL:35.1 ± 0.7 dB, ML‐CL:34.7 ± 0.7 dB. *n* = 8 targets (total 2 animals) per group. (F) Mean (left) and standard deviation (right) of 7^th^ harmonic emission during sonication by ML‐CL. A variance test (*f*‐test) was performed to compare the standard deviation (right) of the mean 7th harmonic emission at each target. (G) MRI T1 images using the controller at 36 dB or equivalent OL pressure. (H) Quantification of *K*_trans_ values through DCE‐MRI. (I) Broadband emission histogram (>6 dB considered an event). 11, 46, 14, and 1 instances of broadband emission event out of 1048 total sonications for *P*_Avg_, *P*_Max_, CL, and ML‐CL, respectively. (J) MLP decision histogram for ML‐CL. 4.4% positive predictions. (46/1040 of sonication). (K) Treatment window analysis for reactive (OL, CL) and predictive (ML‐CL) controllers, showing a ∼300% expansion with ML‐CL (0.09 vs. 0.02 MPa for OL/CL) and a 188% shift in *K*_trans_‐to‐broadband probability curve, indicating improved BBB opening at matched safety. A more detailed figure with individual data points is shown in Figure S8. ns = not significant, ^*^ *p* < 0.05, and ^**^ *p* < 0.01. Statistical analyses were performed through One‐way ANOVA and Bonferroni correction. For the variance test, *f*‐test was used with Bonferroni correction.

In subsequent experiments (Figure 2A), we selected a 7th harmonic target level of 36 dB, which lies beyond the range of the cavitation threshold curve. The absence of representative pressure from the cavitation threshold model (P_Model_ = N/A) indicates that the 36 dB target level presents a challenging condition for all controllers (Figure 2B). Note that while the threshold selected is above the controller calibration curve, it is still within the distribution of the data that we used to train the MLP, which is critical for accurate predictions (i.e., no extrapolating out of distribution). Again, the OL controllers operated at the pressure levels determined by the ML‐CL operation (at 36 dB target level). That was at average (P_Avg_) and maximum pressures (P_Max_) of 0.25 and 0.33 MPa, respectively (Figure 2C). We also incorporated a state of the art closed‐loop controller (CL) [17], operated at 36 dB target level. For each controller algorithm, we assessed the resulting AE, total broadband emission events, and BBB opening strength, as before (Figure 2D,E). While in this condition, we found no significant difference in AE levels achieved among controllers (i.e., presumably due to AE saturation as observed in Figure 2B), closed‐loop control algorithms CL and ML‐CL exhibited significantly lower AE fluctuation compared to the OL throughout the sonication (Figure 2F), which reflects their ability to consistently maintain certain level of MB dynamics. Similar to AE, *K*_trans_ values indicated comparable BBB permeability across all groups (Figure 2G,H), except for *P*_Avg_ and CL. The lack of significant difference between the groups is likely due to the high variance observed in the *K*_trans_ for the OL group, which may stem from its corresponding variability in AE. Importantly, when we assessed the presence of broadband emission events, we found that OL controllers operated at *P*_Avg_ and *P*_Max_ resulted in 1.05% (11/1048) and 4.39% (46/1048) of sonications with broadband emissions, respectively (Figure 2I, orange and red). Interestingly, the CL group also showed a notable presence of broadband emission events in 1.34% (14/1048) of sonications, indicating that 36 dB target level not only represents a limiting condition for OL control algorithms but also for all the controllers (including CL) that react to the broadband events (Figure 2I, orange, red, and green). In contrast, the proposed ML‐CL algorithm recorded only one instance of broadband emission event (1/1048, less than 0.1%), representing a 93% reduction compared to the CL algorithm (Figure 2I, blue). This reduction is likely due to the MLP algorithm's predictions during ML‐CL operation, with 4.4% (46/1040) of its sonications flagged as potential broadband emission events (Figure 2J). This is almost 20‐fold higher prediction rate compared to 32 dB target level (Figure S6I), highlighting the versatility of ML‐CL and its ability to maximize the controller's efficiency while maintaining a strong safety profile. Furthermore, applying MLP to the OL algorithms’ post‐operation AE revealed that it could have predicted 51% of OL's broadband emission events (Figure S7).

#### 2.3.3 ML‐CL Expands Treatment Window

Subsequently, to determine treatment window improvement by ML‐CL, we gathered the sonication data (e.g., pressure and broadband levels) from the reactive (OL, CL—i.e., No ML) and predictive (ML‐CL) controllers and their resulting *K*_trans_ (Figure 2K). We defined 0.17 MPa (P^32dB^_Avg_) as a conservative lower bound of the treatment window, as the *K*_trans_ rose above the background at this pressure (Figure S6G). We then defined the upper bound of the treatment window as the pressure that resulted in a notable increase in broadband emission levels (i.e., point of inflection: 0.19 MPa for reactive controllers; 0.26 MPa for ML‐CL). Strikingly, such pressure‐bound analysis indicated that the ML‐CL expanded the acoustic treatment window by 4‐fold, from 0.02 to 0.09 MPa (Figure 2K, top). Moreover, by performing a similar analysis of *K*_trans_ and broadband emission event probability (Figure 2K, bottom right), we found ML‐CL achieved three‐fold increase in BBB permeability in healthy mice brains, given the same risk of brain injury compared to reactive controllers (Figure 2K, bottom right). Collectively, our results demonstrate that the integration of ML in MB‐FUS mediated BBB opening control algorithms quadruples the treatment window, leading to improved BBB opening for a reduced risk of MB collapse (i.e., maximum benefit/risk ratio).

#### 2.3.4 Safety Confirmation of ML‐CL at High Exposure Condition (36 dB)

**FIGURE 3:**
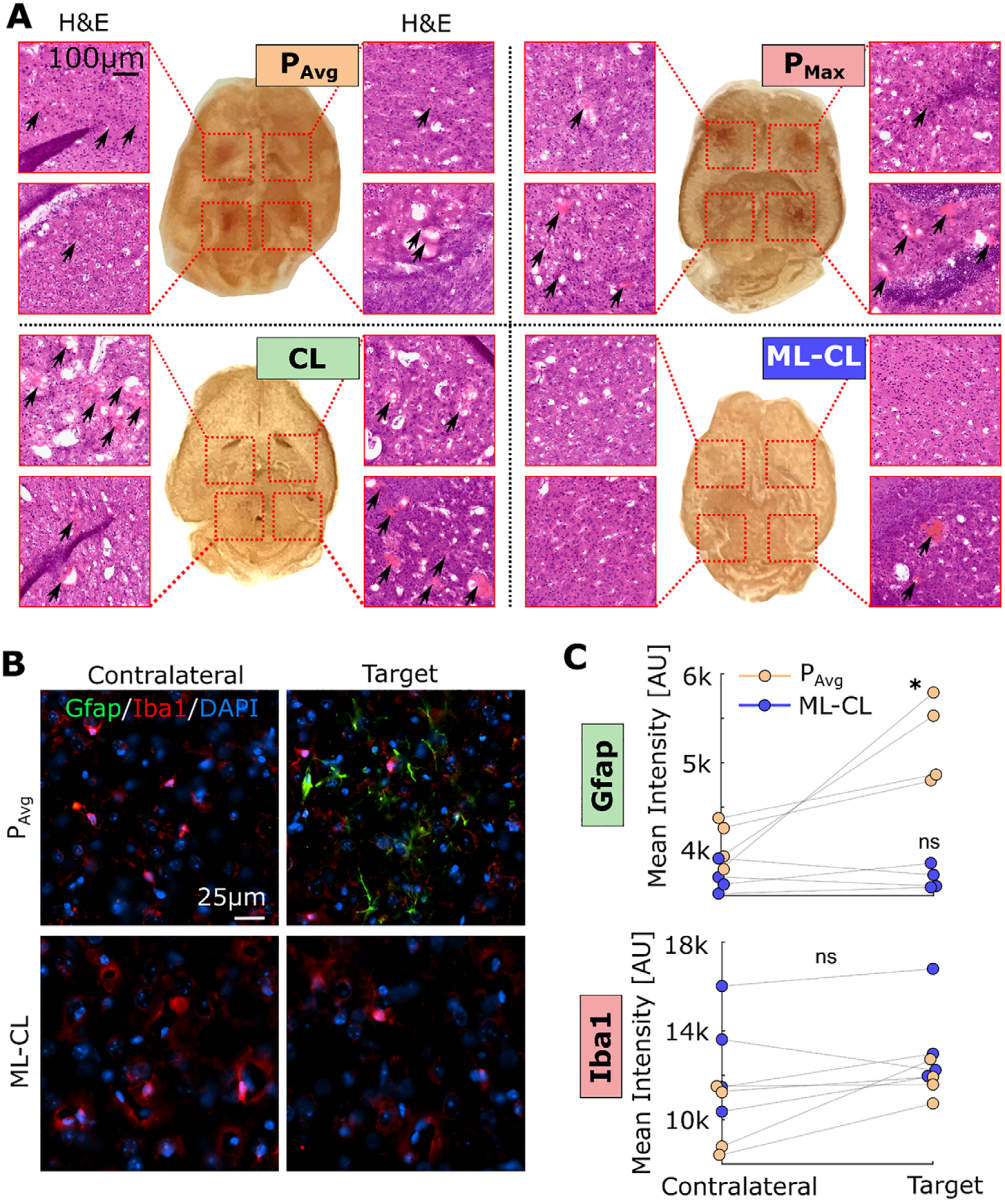
ML‐CL can enhance safety without compromising BBB opening efficiency. (A) H&E staining at each target is referenced in the photos of brain slices in the middle. (B) Representative immunofluorescence images of GFAP and Iba1. (C) Quantification of GFAP and Iba1 intensity comparing the contralateral region and sonicated region. GFAP expression showed a significant increase (^*^ *p* < 0.05) in *P*_Avg_ group but not in ML‐CL. No differences in Iba1 were observed. Paired t‐tests were used for statistical analysis. ns = not significant, ^*^ *p* < 0.05.

Lastly, to directly assess the ability of ML‐CL on preventing tissue damage, we examined the brain tissues sonicated with 36 dB target level using H&E staining (Figure 3A). We found that all the MB‐FUS target locations that were sonicated with the OL controllers (*P*_Avg_ or *P*_Max_) with broadband emission events had either visible or H&E‐evidenced hemorrhages (i.e., petechiae) (Figure 3). Similar responses were observed with the CL group, although we identified petechiae at 2 (out of 8) targets where the broadband emission was undetected (Figure S9). ML‐CL group showed hemorrhage only at one target where a broadband emission event was detected (Figure 3A, bottom right). In a separate cohort, we sacrificed the animals 6 h post sonication and stained the brains for astrocytosis (GFAP) and microglial activation (Iba1), which are established markers for assessing neuroinflammation [45, 62, 63, 64]. We found that compared to the contralateral unsonicated region, *P*_Avg_ group showed an increase in GFAP expression (Figure 3C, top), indicating early signs of astrocytosis in response to sonications. ML‐CL revealed no significant increase in the GFAP expression, highlighting its ability to maintain safety. At this time point, we did not observe differences in Iba1 expression in either group. The observed lower expression of Iba1 compared to GFAP expression is consistent with previous findings [62]. Together, our findings confirm that broadband emission events are key indicators of brain damage and demonstrate that integrating ML (i.e., predictive element) into a closed‐loop controller can persistently and proactively ensure safety and consequently expand (four‐fold) the treatment window of MB‐FUS‐mediated BBB opening.

### 2.4 ML‐CL Augments the Safe and Efficient Delivery of PEGylated Polystyrene Nanoparticles

**FIGURE 4:**
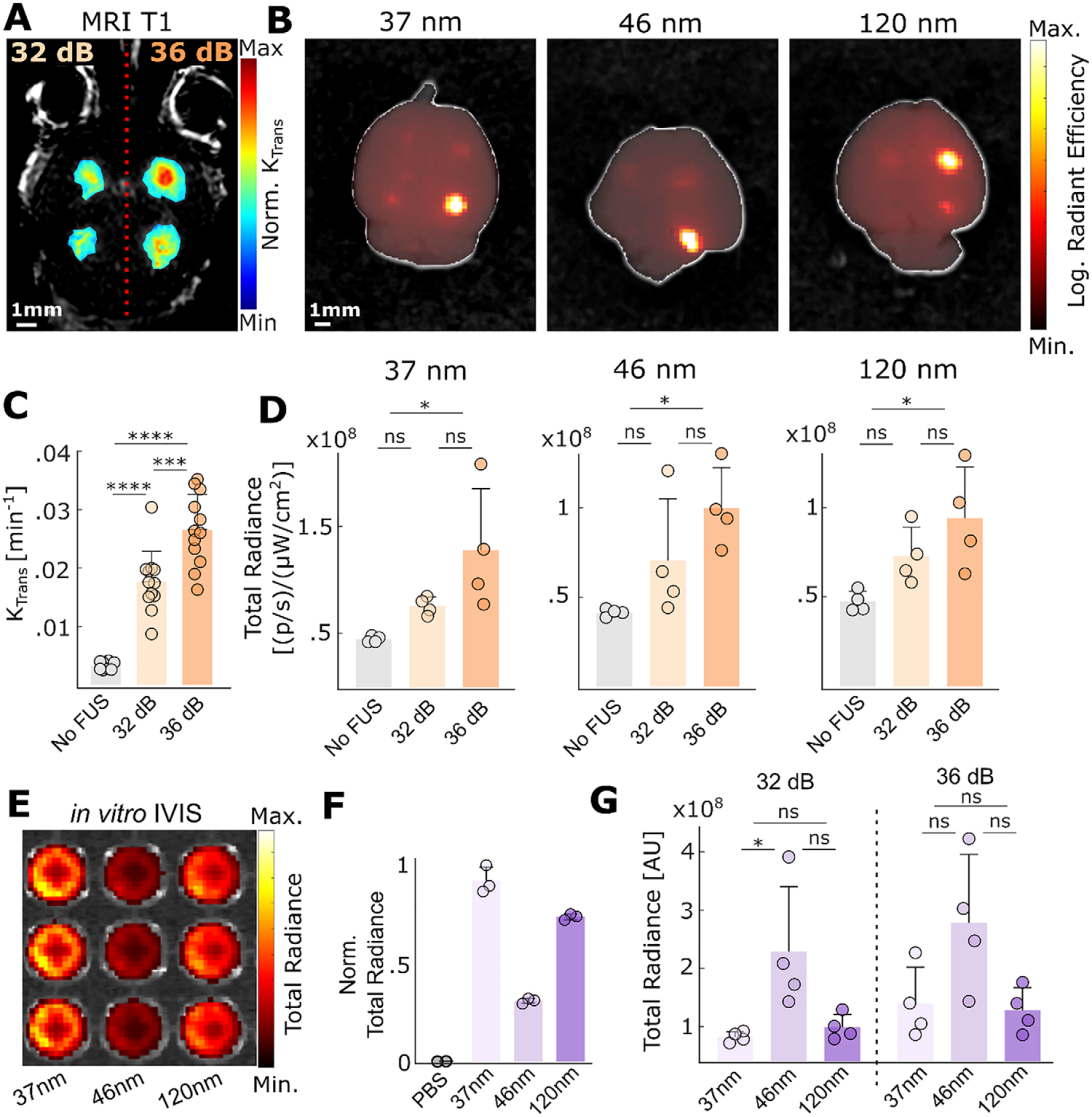
By expanding the treatment window, ML‐CL can safely enhance the delivery of PEGylated polystyrene nanoparticles with broad size distribution in the healthy brain. (A) Representative T1‐MRI image after ML‐CL sonication. Left brain hemisphere was treated with 32 dB, and the right brain was treated with 36 dB ML‐CL. *n* = 2 animals (total 4 targets) per group. (B) Representative IVIS imaging showing delivery of each size NPs at ML‐CL targets. Color range is formulated in a logarithmic scale. (C) Quantification of *K*_trans_ for each target level. Both 32 and 36 dB target levels resulted in significantly higher *K*_trans_ compared to the unsonicated region (*p* < 0.0001). 36 dB group had higher *K*_trans_ compared to 32 dB group (*p* < 0.001). Statistical analysis was performed through one‐way ANOVA with Bonferroni correction. (D) Quantification of IVIS fluorescence for each size NPs. 36 dB group increased 37, 46, and 120 nm NP delivery by 2.8, 2.1, and 2.1‐fold, respectively (^*^ *p* < 0.05). Statistical analysis was performed using one‐way ANOVA with Bonferroni's correction. (E) Representative image of in vitro IVIS setup with the different NPs (injection concentration) on a well plate (see Methods for details). (F) We used the normalized in vitro radiance from each NPs to account for differences in their in vivo fluorescence. (G) Normalized quantification of D using in vitro fluorescence ratio. ns = not significant, ^*^ *p* < 0.05, ^**^ *p* < 0.01, ^***^ *p* < 0.001, and ^****^ *p* < 0.0001. Statistical analyses were performed through One‐way ANOVA and Bonferroni correction.

Following the evaluation of ML‐CL and establishing exposure conditions for robust BBB opening without compromising safety, we assessed the impact of the widened treatment window using ML‐CL to deliver fluorescently labeled PEGylated polystyrene nanoparticles (NPs) of different sizes (37, 46, and 120 nm after PEGylation; detailed characterization shown in Table 4). NPs used in our study serve as well‐defined and reproducible model nanocarriers that enable quantitative assessment of how the ML‐CL influences NPs’ delivery independent of drug‐specific effects [65]. We sonicated healthy mice at one posterior and one inferior region on each brain hemisphere, where left and right hemispheres were sonicated with ML‐CL operating at 32 and 36 dB, respectively (Figure 4A). Immediately after sonication, we intravenously delivered NPs and analyzed the resulting delivery using IVIS (Figure 4B). During sonication (Figure S10A,B), we observed only one instance (1 out of 1048 total sonications) of broadband emission event at 36 dB target level (one target from the 120 nm group, Figure S10C). Hence, we deduced that the safety profile was comparable to our previous investigations (Figure 2 and 3). Moreover, our analysis of *K*_trans_ (Figure 4C) supported previous observations that indicated a target‐level‐dependent increase in BBB permeability in healthy brains. In agreement with the levels in AE and *K*_trans_, we observed enhanced NP delivery by 2.8‐fold, 2.1‐fold, and 2.1‐fold for 37, 46, and 120 nm sizes, respectively (compared to non‐sonicated regions—Figure 4D; 36 dB targets). While the data had significant variation, presumably due to IVIS assessment (i.e., lower detection sensitivity for deeper targets), the NP delivery was significantly higher (*p* < 0.05) only for the 36 dB sonication. This indicates that the improved treatment window by ML‐CL is critical for improving NP (up to 120 nm) delivery in the brain. We also investigated the size dependency of in vivo NP delivery by accounting for size‐dependent total radiance (Figure 4E,F). Interestingly, we found that 46 nm had maximum delivery (Figure 4G). While these data seem counterintuitive, they support past studies that have shown that the delivery of NPs in the range of 40–50 nm is optimal after MB‐FUS [66]. In aggregate, these findings demonstrate the ability of ML‐CL to significantly enhance the delivery of NPs in the murine brains without compromising safety by critically expanding the treatment window of MB‐FUS.

### 2.5 ML‐CL Augments the Release of Brain Cancer Soluble Biomarkers to the Circulation

**FIGURE 5:**
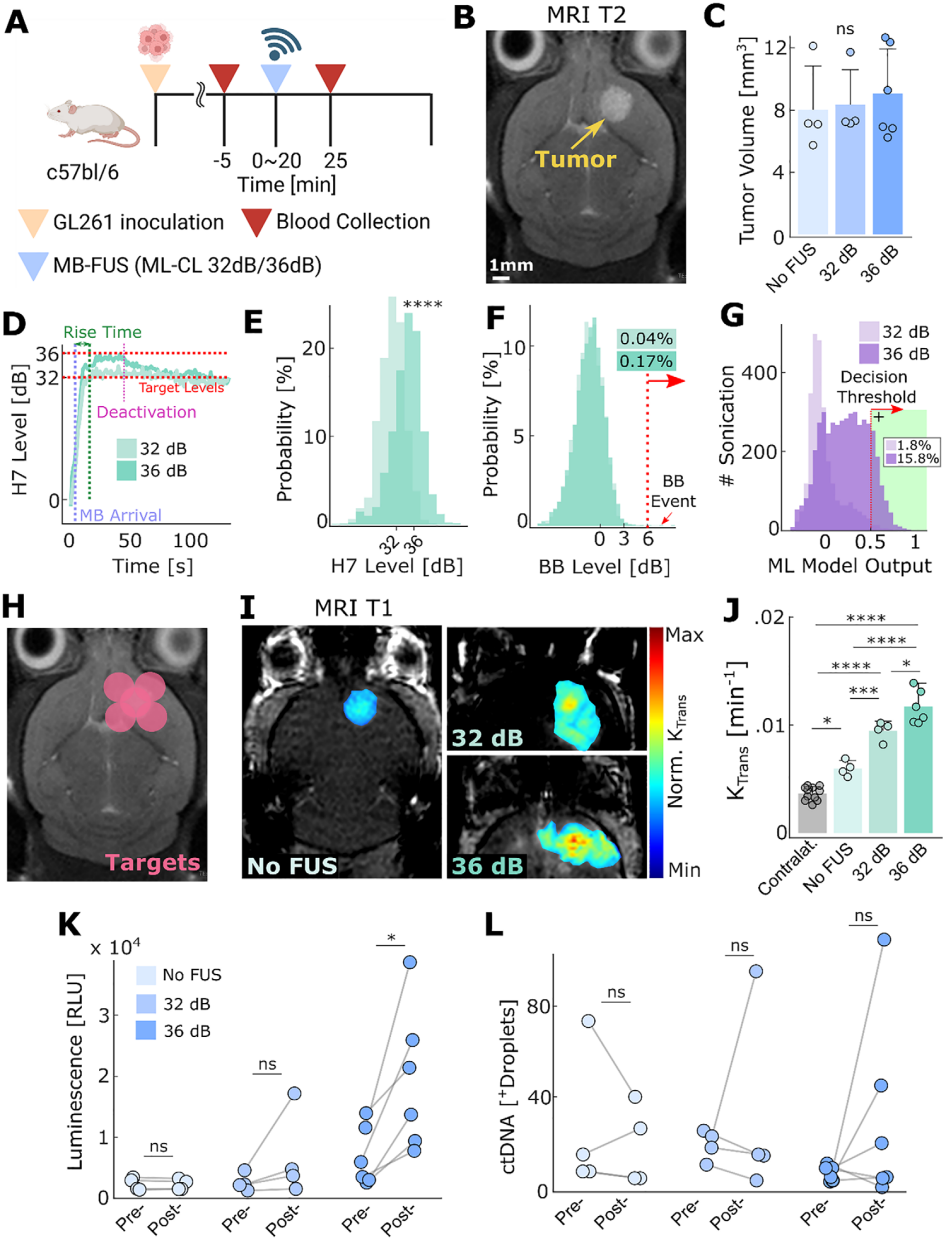
ML‐CL augments the release of brain cancer‐soluble biomarkers. (A) Experimental procedure indicating the time points for treatment and blood collection. The control group was injected with MB only as treatment. (B) Representative T2‐MRI images for tumor, GL‐261‐GLuc. (C) Tumor size distribution across groups. All groups had no significantly different tumor size distribution. (*n* = 4, control, *n* = 4, 32 dB, *n* = 6, 36 dB, respectively). (D) Transient 7th harmonic emission of 32 dB (light green) and 36 dB (green) ML‐CL during its application on GL‐261‐GLuc tumor‐bearing mice. (E) Distribution of 7th harmonic emission for ML‐CL at 32 and 36 dB target levels. 7th harmonic level distribution was compared with *t*‐test. (32 dB—30.96 ± 3.13 dB; 36dB—34.57 ± 3.73 dB). (F) Histogram of broadband emission during sonication. 32 dB ML‐CL had 0.04% (1/2620), and 36 dB had 0.17% (8/4585) of sonication that contained broadband events. (G) ML model output during sonication. The model predicted broadband emission at 1.8% (48/2620) and 15.8% (722/4585) of 32 dB and 36 dB ML‐CL sonication, respectively. (H) ML‐CL target locations. (I) Representative MRI‐T1 images and (J) quantification of *K*_trans_. (K) GLuc protein luminescence quantification. Mean relative change—Control group: no difference (ns), 32dB: 2.2‐fold (ns), 36 dB: 3.3‐fold (*p* < 0.05). (L) GLuc gene quantification. Mean relative change—Control group: no difference (ns), 32dB: 1.5‐fold (ns), 36 dB: 7.8‐fold (ns). ns = not significant, ^*^ *p* < 0.05, ^**^ *p* < 0.01, ^***^ *p* < 0.001, ^****^ *p* < 0.0001. All statistical analyses were performed using One‐way ANOVA with Bonferroni correction. Specific samples were excluded, check methods for exclusion criteria.

Subsequently, using tumor‐bearing GL‐261 mouse models, we assessed the ability of the ML‐CL controller to augment the release of soluble biomarkers into the circulation. In this proof‐of‐concept investigation, we transduced the GL‐261 cell line with Gaussia Luciferase (GLuc), so they can release a luminescent protein [67, 68]. This allows us to concurrently compare the release of GLuc protein (∼20 kDa) and gene (∼60 kDa) [69, 70] with high specificity. We sonicated the GL‐261‐GLuc tumor‐bearing mice using ML‐CL (32 or 36 dB target level) and collected blood pre‐ and post‐treatment (Figure 5A). For non‐sonicated control group (i.e., MB only; No FUS), we followed the same blood collection protocol. We chose 32 or 36 dB ML‐CL target levels based on the NP delivery experiments, where the former represents a conservative exposure (i.e., close to current safety levels) and the latter can consistently improve the transport of large molecules across the BBB (Figure 4). The tumor sizes were evenly distributed among the groups (Figure 5B,C), and treatment groups were sonicated in 5 locations across the tumor; this sonication protocol (multiple‐targets to cover the tumor) was employed in all subsequent experiments involving tumor‐bearing rodents.

Interestingly, we found that the average harmonic target level achieved by ML‐CL (34.57 ± 3.73 dB) was lower than in healthy brain study (35.1 ± 0.7 dB) (Figure 5D,E). While this is somehow surprising, it is nevertheless consistent with our observations during SHAP analysis of the MLP, which suggested that the presence of a tumor drives the model toward positive predictions (Figure 1D), which activates the MLP and forces the controller toward lower pressures and consequently toward lower harmonic levels. In fact, while we saw a similar probability of broadband emissions compared to healthy brain (0.17% vs. 0.1%) (Figure 5F), we also observed an increase in total predictions made by MLP (15.8%) in the presence of a tumor as compared to healthy brain (4.4%) (Figure 5G), further corroborating the SHAP analysis. These results highlight ML‐CL's versatility and robustness to different biological environments of tumor‐bearing mice.

As evidenced by the increase in *K*_trans_, non‐sonicated control tumors were leakier than contralateral healthy brains. However, after ML‐CL operation on tumors (Figure 5H), there was a significant increase in BBB permeability in sonicated tumors as compared to control group tumors (Figure 5I,J). Crucially, we found that only for ML‐CL operating at 36 dB, the GLuc protein concentration in the blood significantly increased (3.3‐fold) immediately after sonication (*p* < 0.05) (Figure 5K). This suggests that, similar to therapeutic agent delivery, release of tumor‐soluble macromolecules requires the higher exposure conditions that only the ML‐CL can attain without compromising safety. Meanwhile, although these GLuc protein levels persisted after 2 h, a declining trend was evident (Figure S11), suggesting a burst release immediately after BBB opening. In contrast to GLuc protein, GLuc gene analysis showed that the levels of positive droplets in the circulation were very low and inconsistent for all groups (Figure 5L), indicating an insufficient amount of ffragments in the collected blood. While the analysis of the smaller and more abundant GLuc protein consistently demonstrated the critical role of BBB permeability in restricting the release of cancer soluble biomarkers, the lack of GLuc gene in our analysis highlighted several challenges in addition to BBB permeability. These are possibly related to the degradation of ctDNA [69] in the circulation, assay sensitivity, or sampling issues related to the relatively low blood volume collection limit allowed for mice. To address the impact of these factors on the detection limit and decipher the role of BBB permeability on ctDNA detection, we decided to conduct experiments in larger animal models (see below).

### 2.6 ML‐CL Improves the Delivery of Nanoparticles in Glioma Tumors in Rats

**FIGURE 6:**
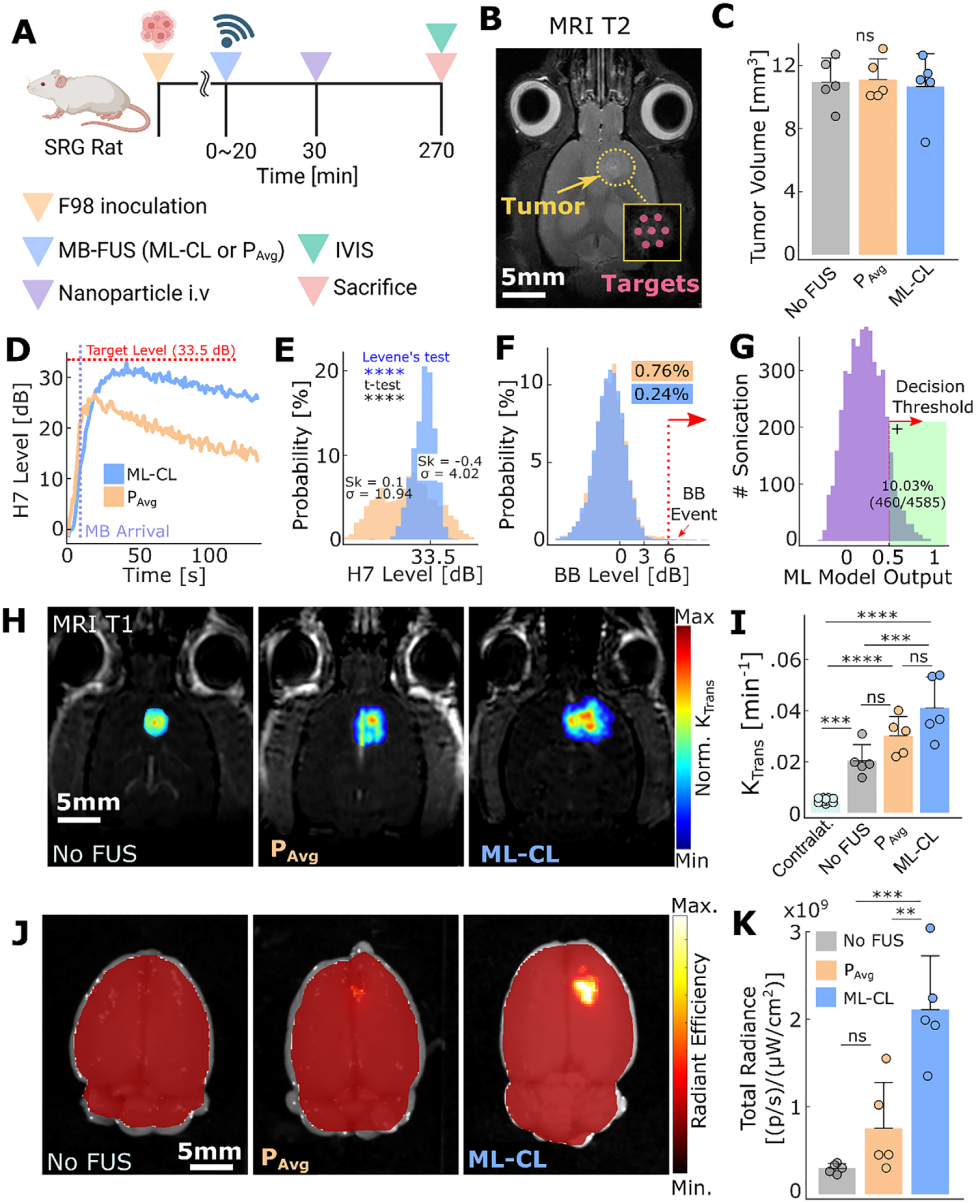
ML‐CL augments delivery of PEGylated polystyrene nanoparticles in F98 tumor‐bearing rats. (A) Experimental procedure indicating the time points for treatment, NP administration, and IVIS analysis. The control group (no FUS) was injected with MB only as treatment. (B) Representative MRI‐T2 image for F98 tumor and sonication target locations. (C) Tumor size distribution across groups. All groups had no significantly different tumor size distribution. (*n* = 5, control, *n* = 5, *P*_Avg_, *n* = 5, ML‐CL 33.5 dB, respectively) (D) Transient 7th harmonic levels during treatment (Blue: ML‐CL 33.5 dB; Orange: *P*_Avg_ 0.29 MPa). Note that the curve is the average of all targets, where MB arrival time and rise time can be different, that led to a lower averaged harmonic level. (E) Histogram of 7th harmonic levels for *P*_Avg_ (Orange) and ML‐CL 33.5 dB (Blue). 7th harmonic levels during 20 s following MB arrival were considered for all sonication. 30.8 ± 4.0 dB for ML‐CL; 24.7 ± 10.9 dB for *P*_Avg_. Levene's test was used for variance comparison and *t*‐test for mean comparison. Sk = skewness. (F) Histogram of broadband emission during sonication. *P*_Avg_ (Orange) had 0.76% (35/4585), and ML‐CL (Blue) had 0.24% (11/4585) of sonication that contained broadband events. (G) ML model output during sonication. The model predicted broadband emission at 10.03% (460/4585) of ML‐CL sonication. (H) Representative MRI‐T1 images and (I) quantification of *K*_trans_. (J) Representative IVIS image of NP delivery in F98 tumor‐bearing rats. Left: Control (no FUS); Middle: *P*_Avg_ (0.29 MPa); Right: ML‐CL (33.5 dB target level). (K) Quantification of NP delivery. ML‐CL improved NP delivery by 7.2‐fold (*p* < 0.001) compared to the control group and 2.8 ‐fold (*p* < 0.01) compared to *P*_Avg_ group. ns = not significant, ^*^ *p* < 0.05, ^**^ *p* < 0.01, ^***^ *p* < 0.001, ^****^ *p* < 0.0001. All statistical analyses were performed with One‐way ANOVA with Bonferroni correction.

To assess the robustness and scalability of the ML‐CL framework and its ability to widen the treatment window, we scaled it up to rats—an inherently more challenging model due to a thicker skull and different MB clearance kinetics—and compared its performance against an open‐loop controller operated at *P*_Avg_. Following the experimental protocols similar to those used in mice (Figure 6A–C), we sonicated F98 tumor‐bearing (SRG) rats with seven non‐overlapping locations with either ML‐CL or *P*_Avg_. For ML‐CL, we conservatively chose a target level of 33.5 dB to account for thicker skulls, and a corresponding *P*_Avg_ of 0.29 MPa was determined from ML‐CL operation (Figure 6B). In these experiments, we were intravenously delivered 46 nm NPs at a dose of 5 mg/Kg after sonication. Note that the minimum operation time for controller (i.e., MB kinetics recording) was increased to 40 s as compared to 20 s in mice to accommodate for the faster MB kinetics in rats.

Interestingly, we observed that compared to *P*_Avg_, ML‐CL achieved a higher mean harmonic level (30.8 ± 4.0 dB vs. 24.7 ± 10.9 dB—Figure 6D,E) and actively shaped its distribution, resulting in a mildly left‐skewed profile (skewness = ‐0.4) with lower standard deviation (10.9 dB vs. 4.0 dB; *p* < 0.0001, Levene's test; Figure 6D‐E). This suggests that in contrast to *P*_Avg_, ML‐CL overcomes weak therapeutic activity and modulates harmonic emissions clustering toward higher values with consistency, thereby highlighting its ability to effectively scale its performance and adapt to different (larger) animal models. Importantly, both controllers maintained comparable and low broadband emission probability (0.76% vs. 0.24% for P_Avg_ and ML‐CL, respectively; Figure 6F). The MLP model exhibited consistent behavior in rats as it did in mice (Figure 6G). In a separate cohort of healthy rats, we confirmed that the ML‐CL exhibits significantly stronger BBB opening (*p* < 0.05) immediately after sonication compared to *P*_Avg_; however, by 24 h the BBB opening level of both sonication protocols was similar (Figure S12) and near baseline levels even after strong opening with ML‐CL (Figure S12). Both *P*_Avg_ and ML‐CL sonications had less than 0.1% (3/2096 and 2/2096, respectively) broadband emission events. Histological analysis of the brains following the sonication indicated no significant differences between *P*_Avg_ and ML‐CL (Figure S13A,B). For both sonication protocols, we observed increased Iba1 signal at the focal region (Figure S13C), which agrees with previous investigations [45, 62, 63, 64]. Collectively our results demonstrate that ML‐CL's safety and performance can be scaled to larger animals alongside its controlling capability.

Our analysis of BBB permeability using *K*_trans_ values corroborated our observations in harmonic level distribution: permeability increased progressively from the control group (untreated) to *P*_Avg_ group and was highest in the ML‐CL group (Figure 6H,I). Surprisingly, despite this trend in *K*_trans_—which also revealed the leaky nature of untreated tumors compared to healthy brain region (Figure 6I)—we observed a notable difference in actual NP delivery. Specifically, NP delivery was negligible in the control group but significantly enhanced only by ML‐CL, achieving a 7.2‐fold and 2.6‐fold increase in delivery compared to the control group and P_Avg_ (Figure 6J,K), respectively. This not only suggests that AEs’ may better predict larger molecule delivery in brain tumors than K_trans_ (i.e., maps the small molecule gadolinium permeability) but also re‐emphasizes the potential of ML‐CL to reliably and safely maximize therapeutic outcomes. Collectively, these findings demonstrate that ML‐CL can be scaled to larger animal models while maintaining its performance (i.e., maximizing the BBB permeability while preventing broadband events) and ability to improve NP delivery in brain tumors.

### 2.7 ML‐CL Augments the Release of ctDNA into Circulation From Glioma Tumors in Rats

**FIGURE 7:**
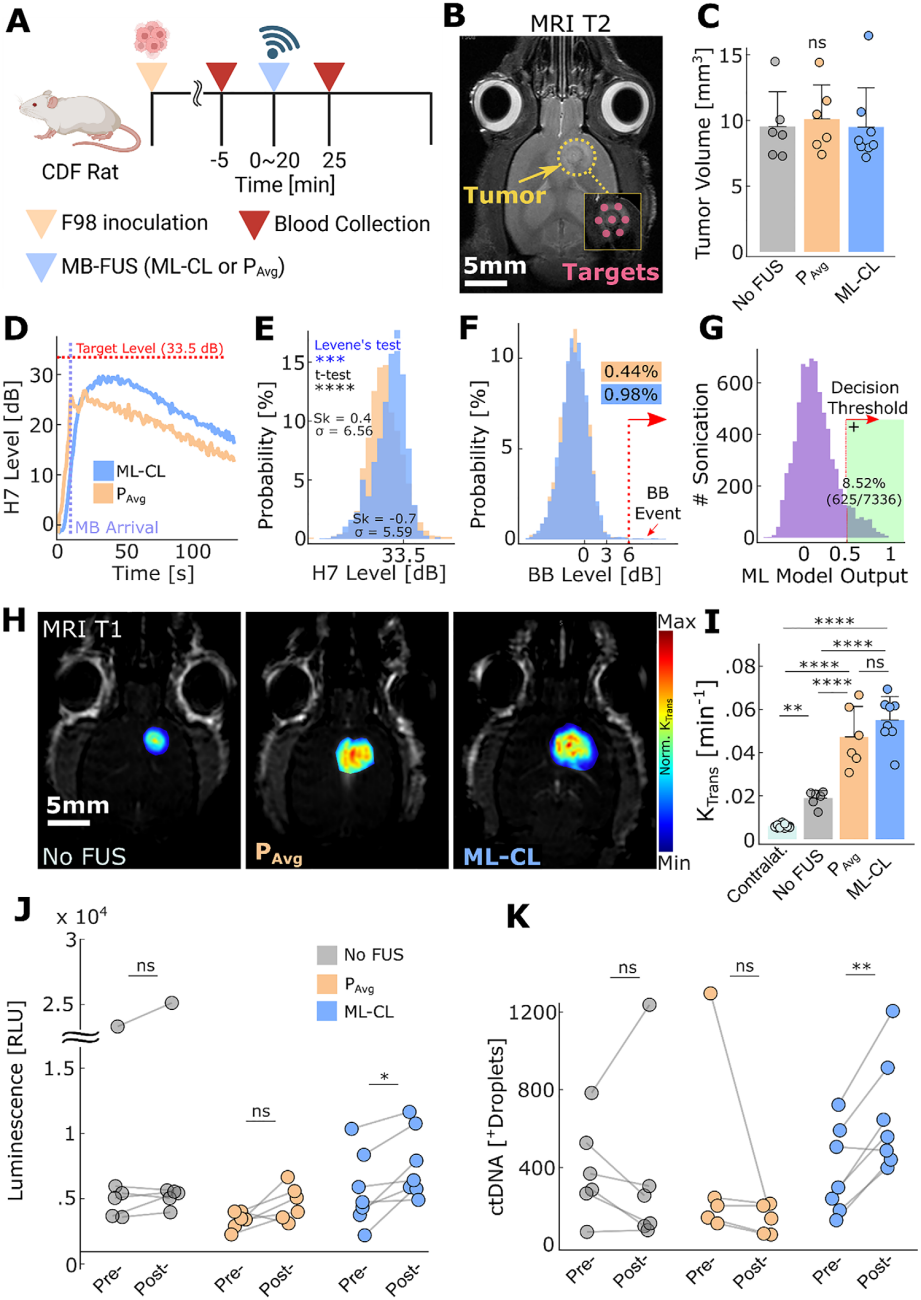
ML‐CL augments the release of protein and ctDNA from F98‐GLuc tumor‐bearing rats. (A) Experimental procedure indicating the time points for treatment and blood collection. The control group (no FUS) was injected with MB only as treatment. (B) Representative MRI‐T2 image for F98‐GLuc tumor and sonication target locations. (C) Tumor size distribution across groups. All groups had no significantly different tumor size distribution. (*n* = 6, control, *n* = 6, *P*_Avg_, *n* = 8, ML‐CL 33.5 dB, respectively) (D) Transient 7th harmonic levels during treatment (Blue: ML‐CL 33.5 dB; Orange: *P*_Avg_ 0.30 MPa). Note that the curve is the average of all targets, where MB arrival time and rise time can be different, which led to averaged down harmonic level. (E) Histogram of 7th harmonic levels for *P*_Avg_ (Orange) and ML‐CL 33.5 dB (Blue). 7th harmonic levels during 20 s following MB arrival were considered for all sonication. ML‐CL: 28.2 ± 5.6 dB and *P*_Avg_: 26.8 ± 6.6 dB. Levene's test was used for variance comparison and *t*‐test for mean comparison. Sk = skewness. (F) Histogram of broadband emission during sonication. *P*_Avg_ (Orange) had 0.44% (24/5502), and ML‐CL (Blue) had 0.98% (72/7336) of sonication that contained broadband events. (G) ML model output during sonication. The model predicted broadband emission at 8.52% (460/7336) of ML‐CL sonication. (H) Representative MRI‐T1 images and (I) quantification of *K*_trans_. (J) GLuc protein luminescence quantification. Mean relative change—Control group: 1.1‐fold (ns), *P*_Avg_: 1.4‐fold (ns), 36 dB: 1.5‐fold (*p* < 0.05). Left: Control (no FUS); Middle: *P*_Avg_ (0.30 MPa); Right: ML‐CL (33.5 dB target level). (K) GLuc ctDNA quantification. Mean relative change—Control group: no difference (ns), *P*_Avg_: no difference (ns), 36 dB: 2.1‐fold (*p* < 0.01). ns = not significant, ^*^ *p* < 0.05, ^**^ *p* < 0.01, ^***^ *p* < 0.001, ^****^ *p* < 0.0001. All statistical analyses were performed with One‐way ANOVA with Bonferroni correction. Specific samples were excluded, check methods for exclusion criteria.

Finally, using F98 tumor‐bearing rat models, we evaluated the ability of ML‐CL controller to improve the release of GLuc protein and gene into the circulation. Compared to mice, rats permit the collection of larger blood samples that allow us to assess its impact on the detection of GLuc protein and gene. In addition to increased blood volume, here we also employed a pre‐amplification step via nested‐PCR technique to further improve the sensitivity of the assay. Similar to our mouse study, we transduced the F98 cell line with Gaussia Luciferase (GLuc) and quantified its protein and gene levels in the blood. As before, we sonicated the F98‐GLuc tumor‐bearing rats using *P*_Avg_ and ML‐CL and collected blood pre‐ and post‐treatment (Figure 7A). For non‐sonicated control group, we injected MB only (No FUS) with the same blood collection protocol. We maintained the tumor‐sonication protocol similar to the NP delivery study (Figure 7B,C). For ML‐CL target level, we chose the same (33.5 dB) as in our NP delivery, where *P*_Avg_ was found to be 0.30 MPa. For these experiments, we chose immunocompetent rat strain (CDF), as it represents a more challenging and clinically relevant model (i.e. rapid clearance and degradation of biomolecules in the circulation). The implementation of the immunocompetent rats leads to lower biomarker concentration baseline [71, 72] (pre‐FUS), which demonstrates a more realistic impact of FUS on molecule release (Figure S14).

Consistent with our previous findings, compared to *P*_Avg_, ML‐CL produced a left‐skewed (skewness = −0.7 vs. 0.41; Figure 7D,E) and tighter (5.6 vs. 6.6 dB standard deviation; *p* < 0.001; Levene's test) harmonic distribution with higher mean (28.2 ± 5.6 vs. 26.8 ± 6.6 dB), reflecting its ability to control and maintain elevated and consistent harmonic levels, as compared to *P*_Avg_. Interestingly, unlike in the NP delivery study, *P*_Avg_ showed reduced harmonic emission variance, presumably due to the smaller size and decreased skull thickness of this rat strain. Nonetheless, our results highlight the inherent shortcomings of open‐loop (constant‐pressure) controllers, which are prone to instabilities in their harmonic emission, such as (i) right‐skewed distribution – likely due to the early influx of MBs after bolus injection – that by nature contributes to lower mean and (ii) higher variability in distribution. Importantly, given that both *P*_Avg_ and ML‐CL showed less than 1% chance of broadband emission event, our results demonstrated that ML‐CL's safety performance is also robust to biological variability introduced by different strains.

Our analysis of BBB permeability using *K*_trans_ values, however, did not reflect our observations in the harmonic level distribution: while permeability increased progressively from the control group (untreated) to the *P*_Avg_ group, ML‐CL group's permeability was not significantly different from *P*_Avg_ (Figure 7H‐I). Surprisingly, despite this lack of trend in *K*_trans_ analysis, which quantifies the permeability of the much smaller MRI contrast agent, we observed a striking difference in the release of the reporter protein and gene, re‐emphasizing the limitation of smaller size molecules (gadolinium) to fully represent the pharmacokinetics of larger size molecules (protein and gene). We observed significant improvement of biomarker release for both protein and ctDNA only for ML‐CL group (1.5‐fold for protein and 2.1‐fold for ctDNA, Figure 7J,K)—note that there was no statistical difference among the baselines (pre‐FUS concentration) of the different groups for both protein and ctDNA. This further underscores the importance of maximizing AE to improve biomarkers’ release—which is an effect that ML‐CL can uniquely achieve while maintaining safety. It also confirmed that the collection of larger blood volumes is critical for effective ctDNA detection. In aggregate, these findings demonstrate that ML‐CL, trained in mice, retains its safety and efficacy in tumor‐bearing rats, hence revealing its translational potential for clinical diagnostic applications with FUS‐enhanced liquid biopsy.

## 3 Discussion and Conclusion

In this work, we developed and evaluated a data‐driven closed‐loop ultrasound nanotheranostic platform for transcranial interventions (Figure 1). The proposed approach critically expanded the current state‐of‐the‐art controllers (OL and CL) by integrating ML to markedly improve the treatment window and overcome rate‐limiting barriers to the delivery of nanoparticles and detection of circulating‐tumor DNA from the brain. Importantly and in relation to the evolving clinical practice [73, 74], our findings indicate that the MB administration protocol is a key safety variable. Even under sonication settings that are generally considered safe under MB infusion, bolus administration can pose a significant risk, as the likelihood of broadband events can be substantially higher during the first pass of MB cloud—an effect that current simple (constant‐pressure) clinical OL controllers cannot easily accommodate [16, 39, 74, 75] unless they dramatically reduce the sonication pressure and by extension the treatment window. Furthermore, OL controllers (constant‐pressure) are prone to inconsistencies in harmonic emissions, including high variability (Figure 6E) and a tendency toward overshooting (right‐skewness; Figure 7E), both of which are intensified by focal pressure uncertainties introduced by the skull. While in that respect, CL controllers are more advantageous by adapting to real‐time AE, they require careful target AE level selection [16, 17, 38], which makes them sensitive to cavitation threshold measurements. They are also bound to low target levels to avoid triggering inertial cavitation and associated broadband emissions (Figure 2I). The proposed data‐driven ML‐CL platform overcomes these limitations. The ability to select target AE level, using a training dataset obtained from past sonications, and the ability to predict broadband events makes the proposed data‐driven ML‐CL ultrasound platform not only less sensitive to these fundamental tradeoffs in controlling MB dynamics but also able to scale to larger animals. Together, these advantages highlight the potential of data‐driven feedback to be integrated into current clinical systems.

While our current ML model was trained using experimental datasets from mice and is therefore specialized for small animal sonications, the underlying framework is inherently scalable. Specifically, the core components of our approach—(1) training a ML model to use sequential sonication data to predict upcoming MB dynamics instability and collapse, and (2) integrating the trained model as an advisory layer to an existing FUS controller (i.e. the specific architecture proposed—see Figure 1C)—are algorithm‐independent and clinically translatable due to their simplicity and flexibility. For clinical implementation, a similar sequential AE dataset can be constructed to train a new model calibrated for human physiology and skull characteristics. We do not view this as a limitation, as current clinical systems already have these data [76]. Moreover, as we showed, the input can incorporate not only AE datasets, but also transient MB kinetics and disease state (e.g., tumor presence). This feature makes the ML‐CL framework amenable to translation, where clinical datasets that include similar multimodal data, such as age, gender, vascularization, disease state, skull porosity (e.g., skull density ratio [77]) can be readily incorporated into the same ML‐CL structure to enable personalized sonication strategies and further optimize clinical MB‐FUS protocols across diverse patient populations. Finally, the framework is not restricted to a specific ML model (e.g., MLP) used in this study. As we demonstrated by successfully training predictive models using alternative architectures, such as attentive deep‐learning MLP (Figure S3), which exhibited less conservative behavior in predicting MB collapse, may therefore be suitable for more aggressive MB‐FUS implementations. Additional ML models can further diversify and enhance data‐driven FUS approaches. For instance, instead of using a single pulse to predict the subsequent pulse, recurrent neural networks could capture longer temporal histories; spectrograms of intra‐pulse AE could be treated as images for convolutional neural network‐based models.

It should be noted that the thicker human skull, together with microbubble (MB) dose–limiting considerations that lead to highly diluted MB infusions, especially when targeting large brain regions [37, 78], may compromise both the bandwidth and sensitivity of recorded acoustic emissions. This is because fewer microbubbles within the focal region generate weaker signals, which are further attenuated as they propagate through the thicker human skull [78]. Although systems with larger focal regions (i.e., higher f‐numbers) and sensitive receivers are expected to produce robust AE signals [79, 80, 81, 82, 83], these challenges must be addressed for effective clinical translation, as ML models require both large and high‐quality datasets.

Despite these challenges, our experimental findings suggest that ML‐CL retains key properties needed for safe and controlled BBB modulation, providing a foundation for assessing its translational potential. Our data indicate that ML‐CL induces a stronger initial BBB opening while maintaining a reversible temporal pattern, with permeability returning close to baseline after 6 h and aligning with constant‐pressure sonification by 24 h (Figure S13). These observations suggest that adaptively modulated sonification does not introduce atypical or prolonged BBB disruption. In addition, the ability of ML‐CL to adjust output based on real‐time acoustic feedback may help mitigate some of the variability and uncertainties associated with fixed‐pressure dosing, potentially supporting more consistent and individualized BBB modulation. Because the controller relies primarily on measurable acoustic emissions and can be further refined using larger and more heterogeneous datasets, the framework may also be adaptable across a range of anatomical and physiological conditions. Altogether, these characteristics suggest that ML‐CL could offer a feasible and safe direction for future clinical translation.

Beyond the clinical implications of the proposed framework, by interpreting the trained MLP model (including additional harmonic components for a broader perspective) it is possible to identify new or important features that shape MB dynamics, using model interpretation techniques such as the SHAP approach. This can be used to enrich existing features for controlling the MB dynamics (e.g., ultra‐harmonics and harmonics). It also revealed that the likelihood of inertial cavitation is greater when sonicating brain tumors, presumably due to higher MB accumulation or possibly due to abnormal vessel structure and mechanical properties. This finding indicates that calibrating the sonication settings in healthy brains and then applying them to diseased tissues can impact the MB dynamics in unexpected ways (inertial vs. stable cavitation) to undermine safety and confound mechanistic investigations that explore specific (immuno)mechanobiological interactions [84]. It further underscores the importance of accounting for biological variability [36, 85] and conducting systematic investigations for establishing treatment windows in disease models [16]. SHAP analysis also revealed that pressure, while being a feature of high importance, its effect on the model's output—hence, MB dynamics—is not monotonic (i.e., clear direction). That is, despite a weakly positive correlation, its effect on the model is likely to be influenced by complex interactions involving other MB AE features. Data‐driven closed‐loop ultrasound can help refine these findings and also support formal parametric analysis aimed at optimizing the FUS pulse sequences (excitation frequency, pulse duration, pulse repetition frequency) and MB properties for promoting desirable bioeffects [58, 86, 87]. Collectively, our study demonstrates that machine learning models and data‐driven analysis can form the basis for studying and analyzing the highly nonlinear and complex nanoscale microbubble dynamics in brain vessels, in addition to allowing for effective control of this highly nonlinear physical phenomenon under in vivo conditions.

Our investigation also demonstrated that the marked improvement in the therapeutic window of MB‐FUS can have important implications for a wide range of therapeutic and diagnostic interventions in the brain. Most notably, the ability to safely deliver a range of NP sizes (36–120 nm) can create new opportunities for targeting brain diseases and potentiate systemically administered novel gene editing nano‐systems that incorporate larger and more potent cargos, thereby allowing them to effectively target a broad range of CNS diseases [21]. Our analysis, which supported prior theoretical prediction that indicated higher delivery for 46 nm NPs under MB‐FUS (Figure 4G) [66], can be further explored to study other NP variables (e.g., surface charge, coating, loaded molecule(s)) that are critical for effective delivery in the brain. Moreover, considering that the AE levels tracked with the delivery of NPs and the release of biomarkers across different species, future applications of ML‐CL technologies could potentially alleviate the need for MRI verification and the associated logistical and financial burdens to the treatments. Thus, AI‐powered ultrasound systems may better support daily/weekly treatments, potentially at an outpatient and/or in limited resource settings, without compromising safety and performance, thereby allowing the rapid and broad dissemination (i.e., similar to US imaging) and effective clinical translation of this transformative technology [16, 28, 43, 44].

The level of robustness and flexibility offered by the developed AI‐powered ultrasound system can potentially be even more important for supporting FUS‐enhanced liquid biopsy, where safety and portability can be crucial [28]. Our study also revealed some important principles for the integration of FUS with liquid biopsy. Most notably, it clearly demonstrated the critical role of BBB permeability in restricting the release of cancer‐soluble biomarkers. It also highlighted that protein‐based biomarkers (i.e., GLuc protein) that are characterized by low molecular size (∼20 kDa) [88] and high biomarker release per cell [89] are easier to detect. While GLuc DNA release was somewhat limited in mice, there are several factors may have contributed to the limited detection of ctDNA in mice. These include: i) the larger size of this molecule (∼60kDa) [69, 70], ii) the low number of copies (2 copy‐limit per cell) [90], and iii) challenges associated with sample collection limit and assay sensitivity [70, 91, 92]. However, as we showed in rats (i) maximizing the treatment window, (ii) improving the assay sensitivity, and (iii) more importantly, increasing the blood collection volume (6.25x larger) are essential for improving ctDNA detection without compromising safety. Collectively, AI‐powered MB‐FUS opens opportunities for repeated sono‐interrogation of brain diseases using ctDNA and may provide a highly efficient and economical way to confirm BBB opening independent from contrast‐enhanced MR imaging. The improved performance observed at larger animals also suggests that translating to humans can potentially lead to better responses, thereby creating further opportunities to genotype brain tumors and longitudinally monitor the biology of therapeutic responses.

Although the MLP used in this study was trained without a validation set or hyperparameter tuning, and therefore the possibility of overfitting cannot be completely excluded, this decision was driven by the considerable diversity across sonications in our dataset. Variations in acoustic propagation paths, skull and tissue properties, MB behavior, and sonication parameters make internal train‐validation splits poorly suited for assessing generalization, particularly for an imbalanced classification task in which positive instability events are relatively uncommon. To maintain model interpretability and limit overfitting under these heterogeneous conditions, we intentionally employed a low‐capacity single‐layer MLP rather than a deeper architecture. Despite these constraints, the model demonstrated strong performance in prospective in vivo experiments that served as an external validation, showing very low false‐negative rates and a conservative false‐positive profile that supports safe sonication. Together, these results demonstrate that ML‐based predictive control is feasible for MB‐FUS interventions, and we anticipate that the framework will continue to improve as larger and more diverse datasets become available.

## 4 Experimental Section and Methods

### 4.1 In Vivo Experiments

All animal procedures were performed according to the guidelines of the Public Health Policy on the Humane Care of Laboratory Animals and approved by the Institutional Animal Care and Use of Committee of Georgia Institute of Technology. 10–14 weeks old female C57BL/6J mice (Jackson Laboratory) were used in this study (N = 37 mice in total, 114 mice in training dataset, and 36 rats in total).

### 4.2 USgFUS System

The system used in this study is a custom‐built portable system that operates in two different modes

#### 4.2.1 Imaging Mode and Treatment Mode

In imaging mode, the system creates a 2D ultrasonic image by raster scanning (30 mm × 30 mm window) using the US imaging probe (Imasonic, 3.5 MHz, −6 dB bandwidth 2.8 MHz) that is coaxially aligned with the FUS transducer. To guide the FUS transducer to a target location in the brain, we overlay the 2D ultrasonic image onto an MRI image using the eyes as a landmark. Desired target coordinates are determined relative to the center of the line connecting the two eyes. After the relocation of the FUS transducer to such target coordinates, a pulse/echo scheme is used to align the ultrasound focus to the desired depth in the brain (relative to the skull) by determining the ultrasound travel time to the skull with sound speed of 1540 m s^−1^. The targeting accuracy of this system is ± 500 µm.

In the treatment mode of the USgFUS system, the imaging probe (see above) operates in passive mode and serves as a passive cavitation detector during BBB opening. When in passive mode, the recorded signal (11ms long) by the transducer is high‐pass filtered (cutoff 0.6 MHz) before it is fed to data acquisition system (Model 5000D, Pico Technology) and analyzed using FFT from the host computer. The FUS transducer (Sonic Concept) is driven by sinusoidal signal (0.5 MHz f_0_; 10 ms pulse length; 1 Hz PRF) generated by a function generator (Picoscope, Pico Technology), which has been amplified by a 50 dB power amplifier (Model 240L, Electronics & Innovation Ltd). The peak negative focal pressure of the FUS transducer in the water (free field) was determined using a calibrated hydrophone (2 mm Model, Precision Acoustics). The frequency bin we used for measuring broadband was 3.61 ± 0.3 KHz (7.22 f_0_), as this was closest to the maximum sensitivity of the PCD (3.5 MHz). The frequency range for ultra‐harmonic and harmonic was determined as 0.5n multiples to f_0_ ± 0.3 KHz (where n is 3,4,5, …).

### 4.3 Design of Closed‐Loop AE Controller for BBB Opening

To attain a consistent level of MB dynamics, we incorporated a closed‐loop controller that we have developed and validated in our previous studies [17]. The controller has three main functions; it (1) mitigates for local AE fluctuation, (2) aims to achieve a target AE level (*L* ^k^_target_; k depicts kth harmonic) of a predefined observer state (*k* = 7 in this study, which we selected from ML feature reduction), and 3) mitigates for global AE decay (MB kinetics).

The controller is also able to track cerebrovascular MB kinetics using the MB tracking pulse, *P*_tracker_. MB kinetics tracking allows an effective time window for controller's operation. To prevent controller's divergence caused by cerebrovascular MB clearance, we employed an MB tracking algorithm that monitors the MB kinetics after they are injected into the animals. The MB tracking pulse is a constant, low‐pressure pulse (0.5 MHz; 10 ms pulse length; 1 Hz PRF; 60 kPa peak negative pressure) that follows the preceding controlled pressure pulse. The pressure for this pulse was chosen by selecting a pressure that resulted in minimal effect on BBB permeability, which was assessed using contrast‐enhanced MRI. We have established criteria for controller's operation: (1) when this MB tracking pulse detects 10 dB rise (relative to background signal) in 4th harmonic (H4) level, controller is turned on; (2) after controller's being turned on, initial slope of MB clearance is monitored for 40 s and is updated in real‐time after the initial recording; 3) when the calculated slope indicates 20% decay from normalized maximum H4 level, the controller is ceased, and therapeutic pulse is maintained at its latest calculated pressure until the end of the sonication (130 s). For consistency, this *P*_tracker_ was also incorporated in all OL controllers in this study that started the controller operation with 10 dB rise in 4th harmonic level.

### 4.4 Machine Learning Model Training and Testing

To establish a machine learning model for broadband emission prediction, a training dataset was formed using past AE data that utilized constant pressure sonication from total of 114 mice (10 ms pulse length, 1 Hz pulse repetition frequency, 0.5 MHz sonication frequency, 130 s sonication). To find the relationship between current timepoint (*t*_n_) sonication to future timepoint (*t*_n+1_) broadband emission, features (i.e., input to machine learning model) were initially selected to be the current (*t*_n_) 1–7th ultra‐harmonic (1.5–7.5f_0_) emission levels, target region in the brain (x and y coordinates referenced to the eyes), pulse number, MB kinetics, pressure, and presence or absence of tumor (Table 1) – 2–8th (2–8f_0_) harmonic emission levels were included for Shapley value (SHAP) analysis. Consequently, a label (i.e., ground truth for supervised classification machine learning) was selected to be binary presence of broadband emission (6 dB above baseline) at the next timepoint's sonication (*t*_n+1_). The training dataset was then formed as N×D matrix, where N is the number of total AE dataset (N = 54040), and *D* is our feature dimension (*D* = 20). The size of the corresponding label was (N×1). All AE levels were in logarithmic values. Our training dataset had a scarce presence of broadband emission above 6 dB, which was 8% (4299/54040) of the total dataset. To train the model under such scarcity, the model training dataset was under‐sampled [52], in which we randomly selected same number of AE data from the absence of broadband emission (label = 0) that matched 80% of the number of broadband emission presence (label = 1). To train MLP model, *feedforwardnet* and *train* functions in MATLAB (Mathworks, MA) were used with 10 neurons in 1 hidden layer (a simple proof‐of‐concept model), epochs size of 1000, and a learning rate of 0.01 [50], using Lavenberg–Marquardt algorithm [51] to minimize mean squared error of the dataset. To test the trained model, the remaining 20% of dataset was used. Model testing was evaluated using a confusion matrix, specifically with accuracy (overall accuracy), sensitivity (i.e., recall, true positive vs. false negative), and precision (true positive vs. false positive), all of which are important metrics to evaluate machine learning models [93]. Feature selection was performed using SHAP values with MATLAB *shapley* function [53], where we selected the top seven features of importance according to the mean of SHAP values for feature reduction.

### 4.5 Attentive Multilayer Perceptron (AMP)

Inspired by attention mechanisms, which have demonstrated the ability to selectively focus on the most relevant features of input data, leading to state‐of‐the‐art performance across multiple domains, including natural language processing [94] and computer vision [95], we extended the MLP concept to context‐aware modeling. The proposed model integrates cross‐attention to allow patient‐specific features (query) to dynamically interact with frequency components (harmonics and ultra‐harmonics) from broadband acoustic emissions (key‐value pairs). This enables the model to selectively prioritize informative spectral biomarkers while suppressing noise, enhancing predictive accuracy for broadband emission, and ensuring a controlled physiological state. The motivation for applying attention is that traditional MLPs treat all input features equally, whereas attention mechanisms facilitate dynamic feature interactions, allowing the model to adaptively weigh critical signals in relation to the individual characteristics. By leveraging cross‐attention, this attention algorithm enables individual non‐MBAE features (e.g., MB Kinetics, tumor presence, etc.) to be processed in the context of real‐time environmental stimuli, allowing for an adaptive decision‐making process to assess MB dynamics are at risk of instability.

The dataset was formed using past AE data that utilized constant pressure sonication from a total of 114 mice. We categorize the input features into two groups: (1) Patient‐specific features, which include the target region in the brain (x and y coordinates referenced to the eyes), pulse number, MB kinetics, pressure, and presence or absence of tumor; and (2) Real‐time treatment feedback, which consists of broadband emission extracted from AE signals. These signals were selected to be the current (*t*_n_) 2–8th (2–8f_0_) harmonic emission levels, 1–7th ultra‐harmonic (1.5–7.5f_0_) emission levels. The labels for supervised machine learning classification were binary indicators of broadband emissions exceeding 6 dB above the baseline at the subsequent sonication (t_n+1_). The training dataset was structured as an *N* × [*D*_1_, *D*_2_] matrix, where *N* presents the total number of AE samples (*N*  =  54, 040), *D*_1_ corresponds to the patient‐specific feature dimension (*D*_1_ =  6), and *D*_2_ represents the real‐time frequency‐driven treatment feedback measurements (*D*_2_ =  [7,  7]). The corresponding labels were stored as an *N* × 1 vector. Since broadband emissions above 6 dB were rare, accounting for only 8% of the total dataset (4299 out of 54 040), we maintained this ratio when splitting the data into training (80%) and test (20%) sets to reflect the real data distribution. To address this class imbalance during training, we employed weighted cross‐entropy loss, assigning a weight of $\left(\frac{54040}{4299}\times \varepsilon \right)$ to broadband emissions exceeding 6 dB classes (in our case, we assign ε  =  2). Additionally, we applied K = 10‐fold cross‐validation in the training dataset to determine the optimal hyperparameters and select the best performance model. To mitigate overfitting, dropout, L2 regularization, and layer normalization were incorporated into the training process.

The AMP architecture is illustrated in Figure S3. In this framework, individual‐specific features first pass through a multilayer perceptron, encoding the information into a latent representation as the query (*Q*), while real‐time frequency emission measurements are processed through another MLP, serving as the key (*K*) and value (*V*). The core of the architecture is the scaled dot‐product mechanism, which enables interaction between these two feature sets. *Q* is derived from subject‐specific features (*f_i_*), which encode the physiological state of the subject before sonication, *f_i_* define the individualized treatment message that the model needs to consider when deciding whether MB dynamics are controlled or at risk of instability. *K* is from real‐time frequency feedback (*f_e_*), reflecting the acoustic response from the AE measurements during the sonication, which contains harmonic and ultra‐harmonic frequency measurements. The role of the *K* is to provide a reference against which the patient‐specific information (*Q*) can be compared. The dot product computation of *QK^T^* produces a score matrix where each information interacts with each measurement in the latent space. After normalization via SoftMax, we can get attention scores that how much focus each individual feature should have on the corresponding real‐time feedback. The *V* matrix contains the actual measured frequency emission information, and multiplying the attention score matrix by *V* produces an adaptive and feedback‐aware feature representation for further classification. The key advantages of AMP lie in its ability to treat subject‐specific and real‐time feedback features separately while enabling context‐aware interactions in the latent space. The attention mechanism employs three learnable transformation matrices, allowing the model to prioritize relevant frequency components dynamically, suppress noise, and thus enhance feature selection automatically. Besides, the designed architecture ensures that the patient's state is continuously re‐evaluated in real time based on AE feedback, improving the accuracy and robustness of broadband emission prediction.

### 4.6 Safety Analysis of Controllers

The performance of the ML‐assisted closed‐loop (ML‐CL) controller was compared with four other controllers during MB‐FUS BBB in healthy mice at two different target levels. The first groups were treated with state‐of‐the‐art closed‐loop (CL) controller [17]. The second and the third group were open‐loop (OL) controllers, and the exposure conditions were selected based on the average and maximum pressure used by ML‐CL (*P*_Avg_ and *P*_Max_). The final group was also open‐loop and used a pressure value from the cavitation threshold model (*P*_Model_) that corresponds to ML‐CL target level. For fair comparison, all controllers (ML‐CL, CL, *P*_Avg_, *P*_Max_, and *P*_Model_) had MB kinetics tracking function (constant 60 kPa pulse, 10 ms pulse length) that initiated the controller operation. As a safety feature, ML‐CL reduces pressure after ML model's prediction of broadband emission, as well as after the occurrence (reaction) of broadband emission; CL and OL controllers reduced the pressure only after the occurrence of broadband emission. To compare the performance of the controllers 32 or 36 dB target levels were chosen from the cavitation threshold model (pressure vs. 7th harmonic level). The first (32 dB) is expected to have a low level of broadband emission, and the second (36 dB) is expected to have a high level. The key features of each controller are summarized in Table 3. For 32 dB operation, ML‐CL (*n* = 2), *P*_Avg_ (*n* = 2), *P*_Max_ (*n* = 2), and *P*_Model_ (*n* = 2), totaling of 12 targets per group. For 36 dB operation, ML‐CL (*n* = 2), CL (*n* = 2), *P*_Avg_ (*n* = 2), *P*_Max_ (*n* = 2), totaling of eight targets per group. Animals from NP delivery were added to the group for *K*_trans_ analysis (six targets per target level). For 36 dB operation in GFAP and Iba1 staining, two targets were sonicated with ML‐CL or *P*_Avg_ on right hemisphere and were compared with the contralateral (left hemisphere).

**TABLE 3:** Different controller algorithms and the control law.

Algorithm name	Pressure	Control law	Safety feature
ML‐CL	Varying	Closed‐loop + MLP	Reaction + Prediction
CL	Varying	Closed‐loop (Chapter 2&3)	Reaction
OL (*P*_Max_)	ML‐CL's Max Pressure (*P*_Max_)	Reduce 5% pressure upon broadband emission	Reaction
OL (*P*_Avg_)	ML‐CL's Average Pressure (*P*_Avg_)	Reduce 5% pressure upon broadband emission	Reaction
OL (*P*_Model_)	Pressure from cavitation threshold model (*P*_Model_)	Reduce 5% pressure upon broadband emission	Reaction

### 4.7 Experimental Procedures

All sonications were performed with the FUS transducer operated at 0.5 MHz, with 10 ms pulse length and 1 Hz pulse repetition frequency, for 130 s under concurrent bolus intravenous (IV) administration of clinical‐grade lipid shell microbubbles (100 µL kg^−1^, Definity, Lantheus Medical Imaging). For rat experiments, lab‐made Definity‐like MBs were used due to limited product supply. MB type, dose, and administration protocols were consistent with those used to collect the machine learning training datasets. During the sonications, we recorded/controlled the AE using the single‐element PCD. Dynamic contrast‐enhanced MRI (DCE‐MRI) was taken right after the sonication sessions. Mouse sonications: For 32 dB target level, six target regions in the brain (three in each hemisphere) were sonicated. For 36 dB target level, four target regions in the brain (two in each hemisphere) were sonicated. For NP delivery, four target regions were sonicated (32 dB on the left and 36 dB on the right). For brain tumor liquid biopsy, we performed five sonications (*xy* directions, separated by 1 mm) to cover the entire tumor and its periphery, and one healthy contralateral target was sonicated for targeting confirmation. Rat sonications: For NP delivery in tumors and brain tumor liquid biopsy, seven target regions were sonicated (*xy* directions separated by 1 mm) to cover the entire tumor and its periphery.

### 4.8 BBB Opening Reversibility and Long Term Safety (48 h) in Rats

To investigate the reversibility of BBB opening using ML‐CL and a longer‐term safety, we sonicated healthy rats (*n* = 4) with 4 targets – 2 targets on the right hemisphere with ML‐CL and 2 targets on the left hemisphere with equivalent average constant pressure (*P*_Avg_, 0.29 MPa). We then assessed BBB opening duration by collecting DCE‐MRI (*K*_trans_) datasets at 15 min, 6 h, and 24 h post‐sonication (Figure S12), followed by IHC (GFAP, Iba1, and Hematoxylin‐eosin) at 48 h post‐sonication (Figure S13). At each of time points, we initially performed MR‐T1 imaging without a contrast agent (to confirm its clearance and eliminate the chances of contrast agent buildup in the brain) followed by DCE‐MRI and another MR‐T1 acquisition.

### 4.9 Dynamic Contrast‐Enhanced Magnetic Resonance Imaging (DCE‐MRI)

To access the vessel permeability in the brain, we measured the volume transfer constant, *K*_trans_, by performing DCE‐MRI (*Pharmascan 7T, Bruker*, IR, echo time, 2.5 ms; rep time, 1019.6 ms; flip angle, 30; FOV, 20 × 20 mm^2^ for mice, 35 × 35 mm^2^ for rats) after the last sonication. Before injecting the contrast agents, we acquired a background image in six different flip angles (2°, 5°, 10°, 15°, 20°, and 30°) to quantify the T1 relaxation map, which is used for *K*_trans_ quantification. Consequently, we started collecting DCE‐MRI with concurrent bolus administration of 0.04 and 0.32 mL gadolinium contrast agent (2 mL kg^−1^, Prohance) for mice and rats, respectively. The collected DCE‐MRIs datasets were analyzed, and *K*_trans_ values were calculated in Horos, using DCE tool plugin (Kyung Sung, Los Angeles, California). The arterial input function was obtained based on Fritz‐Hansen et al. method, as provided in the plugin [96].

### 4.10 Brain Tissue Processing

Following MB‐FUS treatments/sonications, the mice and rats were euthanized 2‐, 4‐, or 48 h post‐treatment and transcardially perfused with 20 and 160 mL of saline, respectively. For brain staining, perfusion continued with 4% paraformaldehyde (PFA) before harvesting the brains. Then, the brains were fixed with 4% PFA overnight at 4°C, followed by immersion in a 30% sucrose solution at 4°C until they sank to the bottom of the container. The brains were then embedded in O.C.T. compound and rapidly frozen to −80°C. Subsequently, 20 µm sections were cut using a cryostat (Leica 3050 S Cryostat) for further analysis. For NP delivery, brains were harvested after transcardial perfusion with saline and analyzed immediately (no exposure to PFA).

### 4.11 Immunofluorescence / Immunohistochemistry Staining and Microscope Imaging

To assess the safety and the biological effects induced by MB‐FUS, immunofluorescence staining was performed on brain tissue slides. Tissues were first fixed in 4% paraformaldehyde at room temperature for 10 min. For sections requiring staining of intracellular markers – GFAP (Abcam ab194324 – clone EPR1034Y) and Iba1 (Abcam ab225261 – clone EPR16588) – which are markers for astrocyte and microglial activation for safety assessment), they were subsequently permeabilized with 0.1% Triton X‐100 in PBS for 5 min. After washing with PBS, the sections were blocked for 1 h at room temperature in a solution containing 1% Bovine Serum Albumin and 5% goat serum in PBS.

The sections were then incubated with the primary antibody of interest, diluted in 1% Bovine Serum Albumin (1:100), for 12 h at 4°C. Following primary antibody incubation, the sections were incubated with the secondary antibody, diluted in 1% Bovine Serum Albumin (1:250), for 1 h at room temperature. To stain the cell nucleus, samples were incubated with DAPI diluted in PBS (1:1000) for 10 min after washing. Finally, the sections were rinsed with PBS to remove excess antibody, mounted with mounting medium, and covered with coverslips. Samples were cured with mounting medium for 24 h in the dark at room temperature before imaging. The sections were imaged with a 20x objective using a fluorescence microscope (Eclipse Ti2, Nikon). The quantification of the fluorescence images was performed using ImageJ.

Hematoxylin‐eosin staining was performed to examine tissue damage and safety. Twenty‐micrometer thick frozen sections (Leica 3050 S Cryostat) were dehydrated beforehand and stained using a Leica Autostainer (ST5010). The sections were imaged with a 20x objective using a brightfield microscope (Eclipse Ti2, Nikon).

### 4.12 Statistical Analysis

Results are expressed as means ± standard deviation (or SEM if stated under caption). Statistical analyses were performed using MATLAB and GraphPad Prism 7.0. For statistical analysis of two independent groups, an unpaired Student's *t*‐test was applied. For statistical analysis of self‐comparison groups, a paired Student's *t*‐test was applied. For grouped analyses, we performed a One‐way ANOVA, multiple comparison with Bonferroni's correction. *p* < 0.05 was considered statistically significant (^*^ *p* < 0.5, ^**^ *p* < 0.01, ^***^ *p* < 0.001, and ^****^ *p* < 0.0001, ns = not significant). For variance tests, *f*‐test were used, and Levene's test was used for skewed distributions. Data distribution was assumed to be normal (unless described in the manuscript for skewed distributions), but this was not formally tested in all data. Tumor‐bearing animals were inoculated with tumors and then randomized before allocation to experimental groups. For the remaining studies, randomization was not relevant, as these experiments were conducted on uniform biological materials, such as commercially sourced cell lines. The investigators were not blinded to group allocation during experiments and outcome analysis. Blinding was not feasible for both the in vitro and in vivo studies, as these experiments were conducted by individual investigators who were aware of the experimental groups. Additionally, blinding was not considered relevant, as the data analysis was performed quantitatively (e.g., bioanalyzer quantification, PCR, and digital‐PCR) and did not involve subjective, qualitative assessments. Animals were randomly assigned to experimental groups, but the experiments were not randomized, and the investigators were not blinded to group allocation or outcome assessment. Animal sample exclusion was minimized and only applied either when critical procedures failed or data fell below or above the negative or positive controls, respectively (see animal exclusion criteria section).

### 4.13 Sample Exclusion Criteria

One mouse was excluded from the 36 dB LB group due to mistargeting of the tumor, confirmed by contrast‐enhanced MR imaging and observed in abnormal fluctuation of biomarker levels in the blood. Protein samples from 1 ML‐CL rat were excluded due to luminescence signal below the negative control, included in the same Bioanalyzer run. ctDNA samples from 1 Pavg rat were excluded as quantification surpassed the positive control included in the same dPCR run. ctDNA samples from 1 ML‐CL rat were excluded due to human error during the purification step. Overall, additional animals were placed in each of these groups to maintain *n* ≥ 5.

### 4.14 Nanoparticle Preparation and Characterization

To formulate PEGylated polystyrene nanoparticles, 20, 40, and 100 nm carboxylate‐modified FluoSpheres (PS‐COOH, Ex/Em: 580/605 nm, Invitrogen) were covalently modified with amine‐terminated 5 kDa MW polyethylene glycol (methoxy‐PEG5k‐amine, Creative PEGWorks) by EDC carbodiimide chemistry, following a protocol we previously described [97, 98, 99]. Briefly, PS‐COOH nanoparticles (2 mg) were mixed with methoxy‐PEG5k‐amine (4–5x equivalent to total COOH groups on surface of PS‐COOH particles) in 100 mm borate buffer (pH 8.2), followed by addition of excess sulfo‐NHS (∼24x), and EDC (∼8x). Particle suspensions were placed on a rotary incubator, and the reaction was allowed to proceed for 4 h at 25°C. After the reaction, particles were purified by ultracentrifugation (Amicon Ultra‐15 mL 100 kDa MW cut‐off, Millipore Sigma) with sterile PBS (pH 7.4) as the buffer (3 washes total). Finally, the purified nanoparticles were resuspended in sterile PBS (1 mL) and stored at 4°C for subsequent characterizations and use in in vivo studies.

The physicochemical characteristics of PEGylated polystyrene nanoparticles were measured in 15x diluted PBS (∼10 mm NaCl, pH 7.4) at 25°C. Hydrodynamic diameter and ζ‐potential (surface charge) were determined by dynamic light scattering and laser Doppler anemometry, respectively, using a Zetasizer NanoZS (Malvern Instruments, Southborough, MA). Particle size measurement was performed at 25°C at a scattering angle of 173° and is reported as the number‐average mean. The ζ ‐potential values were calculated using the Smoluchowski equation and are reported as the mean ζ ‐potential. Detailed characterization is shown in Table 4.

**TABLE 4:** PEGylated polystyrene nanoparticle characterization.

	Pre‐pegylation				Post‐pegylation			
NP Size	Z‐Average Size (nm) ± Stdev (*n* = 3)	Number Average Size (nm) ± Stdev (*n* = 3)	PDI (0‐1) ± Stdev (*n* = 3)	Zeta Potential (mv) ± Stdev (*n* = 3)	Z‐Average Size (nm) ± Stdev (*n* = 3)	Number Average (nm) ± Stdev (*n* = 3)	PDI (0‐1) ± Stdev (*n* = 3)	Zeta Potential (mV) ± Stdev (*n* = 3)
**37 nm** **(20 nm PS‐COOH)**	35.83 ± 3.79	22.97 ± 2.48	0.11 ±0.02	−28.77 ± 4.09	56.19 ± 7.64	36.94 ± 4.73	0.14 ± 0.01	−2.91 ± 1.21
**46 nm** **(40 nm‐PS‐COOH)**	51.95 ± 5.27	34.9 ± 3.75	0.10 ± 0.06	−28.23 ± 3.22	60.85 ± 4.19	46.13 ± 2.54	0.06 ± 0.02	−2.04 ± 1.03
**120 nm** **(100 nm‐PS‐COOH)**	129.77 ± 4.12	108.60 ± 3.41	0.03 ± 0.01	−41.43 ± 1.63	139.83 ± 2.43	119.87 ± 2.18	0.01 ± 0.01	−2.64 ± 1.42

The PEGylated polystyrene (PEG‐PS) nanoparticles used in this study were employed as standardized, size‐defined model carriers rather than as therapeutic platforms. This choice enabled reproducible and quantitative assessment of how the ML‐assisted closed‐loop (ML‐CL) controller influences nanoparticle transport across the blood–brain barrier (BBB), independent of drug‐specific chemistry or degradation effects. Polystyrene cores provide exceptional monodispersity, chemical inertness, and stable fluorescence, making them an established surrogate system for evaluating the physical determinants of focused‐ultrasound‐mediated BBB permeability. By using PEG‐PS nanoparticles with well‐characterized hydrodynamic diameters (37, 46, and 120 nm), narrow polydispersity (PDI < 0.15), and near‐neutral ζ‐potential (−2–−3 mV), we could systematically examine the relationship between particle size and FUS‐enhanced delivery efficiency under identical acoustic conditions. This approach isolates the performance of the ML‐CL control framework itself and establishes a reproducible experimental reference that can be directly extended to therapeutic nanocarriers of comparable size and surface characteristics.

Carboxylate‐modified polystyrene nanoparticles were PEGylated using EDC/sulfo‐NHS carbodiimide chemistry to form covalent amide bonds between surface ─COOH groups and methoxy‐PEG‐NH_2_ (5 kDa), following previously published protocols [65, 97, 99]. PEG surface density and conformation were characterized in prior work by ^1^H‐NMR and shown to yield dense brush‐regime coatings (Γ/Γ ≥ 2; ∼9–16 PEG chains/100 nm^2^)* with near‐neutral ζ‐potentials (−2–−5 mV). The same formulation chemistry and coating parameters were used here to ensure reproducible nanoparticle diffusivity within brain tissue.

The PEGylated nanoparticles used here were synthesized using the same EDC/sulfo‐NHS coupling of 5 kDa PEG‐NH_2_ to COOH‐terminated cores previously shown to enable robust diffusion through human and rodent brain ECS [65] and deep parenchymal distribution in glioma tissue [97]. Dense PEG brush coatings (Γ/SA ≥ 2; ζ ≈ −2–−3 mV) and particle sizes < 120 nm fall within the established thresholds for efficient tissue penetration [65, 99]. Accordingly, the increased BBB permeability achieved by ML‐CL is expected to be followed by effective nanoparticle dispersion within brain parenchyma, consistent with these prior findings.

### 4.15 Nanoparticle Delivery and Quantification

The sonications were performed as described previously, but the order of the first sonicated side was alternated in between mice of the same group to account for MB accumulation. After the sonications were completed, each mouse received a 100 µL intravenous bolus‐injection of the designated NP size. PEGylated NPs were prepared for these injections at a concentration of 5 mg kg^−1^. Considering an average body weight of 20 g for mice and 160 g for rats, each mouse received 0.1 mg diluted in 100 µL and each rat received 0.8 mg diluted in 800 µL. The 37, 46, and 120 nm‐NPs had stock concentrations of 1.3, 1.8, and 4.0 mg mL^−1^. For mice, considering a 10% additional volume preparation, 84.61 µL of 37 nm‐NPs were mixed with 25.39 µL of saline, 61.11 µL of 46 nm‐NPs were mixed with 48.89 µL of saline, and 27.5 µL of 120 nm‐NPs were mixed with 82.5 µL of saline, making a total of 110 µL of prepared NPs per injection. For rats, considering a 10% additional volume preparation, 676.9 µL of 37 nm‐NPs were mixed with 203.1 µL of saline, 488.9 µL of 46 nm‐NPs were mixed with 391.1 µL of saline, and 220 µL of 120 nm‐NPs were mixed with 660 µL of saline, making a total of 880 µL of prepared NPs per injection. Animals were euthanized 4 h‐post NP injection and transcardially perfused with 20 mL (mice) or 160 mL (rats) of saline.

To account for variations in size‐dependent NP fluorescence signal, in vitro NP signal was assessed a priori for each size using the concentrations detailed above. Triplicate samples of 55 µL of PBS and each NP size, same concentration from in vivo experiments, were individually placed into a black‐walled, clear‐bottom 96‐well. Then, the well plate was placed in the Perkin Elmer IVIS Spectrum CT (Waltham, MA, USA) for fluorescent imaging of the brain. The machine was programmed using the Perkin Elmer Living Image 4.7.4 to capture fluorescent images with excitation at 570 nm and emission at 620 nm, exposure time of 0.5 s, lamp at low level, medium binning, C field of view, subject height at 1.00 cm, chamber temperature at 37°C, and the lamp temperature at −90°C. The machine was initially programmed to match the fluorescent particle's original excitation of 580 nm and emission of 605 nm, but it automatically defaulted to the values above due to the range of filters installed. Measurements of total radiant efficiency were taken by placing the uniform region of interest (ROI) at the center of each well and covered the entire surface area. The color scale range was set from 2 × 10^9^ to 5 × 10^10^ (p s^−1^)/(µW cm^−2^). The in vitro samples were used to normalize the fluorescent signal from 0 to 1 (the highest measurement). Once normalized, the mean ratio of each size group was calculated and used to correct the recorded in vivo levels, as described below.

The harvested brains were immediately placed in the Perkin Elmer IVIS Spectrum CT (Waltham, MA, USA) for fluorescent imaging. The machine was programmed as before with exposure time of 10.0 s. The fluorescent image overlapped with the photograph of the brain. Measurements of total radiant efficiency were taken by placing the uniform ROI – width and height equal to 0.55 cm – at the center of each sonication target. The color scale range was set from 9 × 10^7^ to 2.5 × 10^8^ (p s^−1^)/(µW cm^−2^). To compare the delivery of NPs, the in vivo data were divided by the mean of the normalized in vitro data of each NP size group.

### 4.16 Cell Lines and Cell Culturing

GL261 glioma (Caliper Life Sciences, Hopkinton, MA, USA) and F98npEGFRviii cells (ATCC, Manassas, Virginia, USA) were separately cultured in Dulbecco's modified Eagle's medium supplemented with 10% fetal bovine serum (FBS) and 1% penicillin‐streptomycin at 37°C and 5% CO_2_.

### 4.17 Gaussia Luciferase Transduction of GL‐261 and F98‐EGFRviii Cell Lines

GL‐261 and F98‐EGFRviii cell lines were transduced with Gaussia Luciferase (GLuc) using polybrene and the lentivirus cytomegalovirus humanized‐GLuc (LV‐CMV‐GLuc‐Puro, SignaGen Laboratories, Frederick, MD, USA) with the multiplicity of infection (MOI) equal to 2. Transduced cells were selected with overnight puromycin exposure at 10 mg mL^−1^.

### 4.18 Tumor Inoculation and Growth in Mice

GL‐261‐GLuc (mice) and F98‐GLuc (rat) cells (10^5^ cells) were stereotactically implanted into the brain at 1 mm anterior, 1  mm to the right of the bregma, and 3 mm deep to skull bottom of 12‐week‐old female C57BL/6J mice (The Jackson Laboratory), or SRG (Sprague Dawley‐Rag2 Il2rg/HblCrl)/Fisher CDF (F344/DuCrl) rats (Charles River). All tumors were allowed to grow up to 2.5 mm, and then mice were evenly distributed among groups according to tumor volume, which was quantified using the ellipsoid volume equation.

### 4.19 Liquid Biopsy Experiments

To assess the diagnostic potential of the proposed ML‐CL controller, we employed it on GL‐261‐GLuc tumor‐bearing mice (*n* = 6) and F98‐GLuc tumor‐bearing rats (*n* = 7). Each animal was treated with 5 (mouse) or 7 (rat) sonication targets using ML‐assisted controller to cover the tumor region at target levels established to be safe in healthy brains. To compare the role of FUS in brain tumor liquid biopsy, we split animals from the same cohort into a control group (*n* = 4 for mouse and 6 for rats), where no FUS was employed. These animals received the same MB injections with time intervals in between to represent each target sonication duration.

### 4.20 Blood Collection and Serum Isolation

For mouse, blood samples (180 µL/each) were collected retro‐orbitally 5 min before and 15 min after sonication of the treatment midpoint (for both FUS and control groups), and 2 h‐post‐treatment (terminal – for FUS group) using EDTA‐coated capillary tubes attached to a non‐coated 1.5 mL microcentrifuge tube. For rats, blood samples (1.2 mL/each) were collected from the subclavian vein 5 min before and after sonication at the treatment midpoint (for both FUS and control groups), using a 23G‐needle attached to a Luer slip syringe, and transferred to a non‐coated 1.5 mL microcentrifuge tube. All samples were allowed to coagulate in ice for 10 min prior to 1000 g centrifugation for 20 min. Serum was allocated for protein quantification (20 µL – all animals) and ctDNA (80 µL for mice and 500 µL for rats) purification/quantification.

### 4.21 Protein Quantification

Serum (20µL) of each sample was placed in a single well of a black‐walled, clear‐bottom 96‐well plate. Coelenterazine solution from the Pierce Gaussia Luciferase Glow assay kit (ThermoFisher, Waltham, MA, USA) was added to each well immediately prior to measurements using Synergy HT Microplate reader (Biotek Instruments, Santa Clara, CA, USA). Luminescence measurements were taken for 10 min at 30 s intervals, with no lid, 1.0 mm height, 100 ms delay, 135 dB gain, and hole emission. Measurements for the first 5 min were considered and integrated, remaining measurements were disregarded due to signal decay after this timepoint.

### 4.22 Circulating‐Tumor DNA (ctDNA) Purification and dPCR Quantification

Serum samples were placed in individual sterile 1.5 mL microcentrifuge tubes, purified using the Plasma/Serum cell‐free DNA Purification kit (Norgen Biotek, Ontario, Canada), and eluted to a final volume of 30 µL. All samples were frozen overnight at −20°C.

Rat samples were pre‐amplified using the Q5 Reaction kit (New England Biolabs, Ipswich, MA, USA) and forward and reverse primers, eluted in 10µm, with sequences 5’‐AGAGATGGAAGCCAATGCCC‐3’ and 5’‐GTGCAGTCCACACACAGATC‐3’, respectively. Each reaction included 10 µL of the Q5 Reaction Buffer and 10 µL of the Q5 High GC Enhancer (B9027S), 0.5 µL of the Q5 High‐Fidelity DNA Polymerase, 1µL of the Q5 dNTP Solution Mix, 0.5 µL of each of the forward and reverse primers, 22.5 µL of PCR‐grade water, and 5 µL of the template DNA sample. Reaction samples were placed in 0.5 mL Eppendorf PCR tubes and run using Applied Biosystems’ Pro Flex PCR Thermocycler, which was programmed to process 1 cyle at 98°C for 30 s, and 10x cycles of 98°C for 10 s plus 68°C for 20 s plus 72°C for 15 s, and 1 cycle of 72°C for 2 min.

Custom fluorescent probe and sequence primers were synthesized for GLuc gene detection (Integrated DNA Technologies, Coralville, IA, USA). The probe, eluted at 100 µm, has sequence of 5’‐/56‐FAM/CTGTCCCA/3IABIFQ‐3’, and the forward and reverse primer sequences for GLuc gene, both eluted at 10 µm, are 5’‐ACCAGGGGCTGTCTGATCT‐3’ and 5’–GGGATGAACTTCTTCATCTTGG–3’, respectively. Mouse and pre‐amplified rat samples were processed for amplification and quantification. Each DNA sample was prepared by mixing 4 µL of template DNA with 1.6 µL of the probe, 0.32 µL of forward and reverse primers, 10 µL of QIAcuity dPCR Probe Master Mix (QiAgen, Hilden, Germany), and 23.76 µL of PCR‐grade water. All dPCR runs included in vitro positive control and negative control (PCR‐grade water in place of DNA) samples. Samples were added to QIAcuity Nanoplate 26k 8‐well/24‐well plates and processed using QiAgen QIAcuity One digital‐PCR (QiAgen, Hilden, Germany). dPCR was programmed to process FAM signal and included 1 cycle at 95°C for 2 min, and 40 cycles composed of 95°C for 15 s and 60°C for 30 s.

## Author Contributions

H.L., V.M., and C.A. contributed in project conceptualization and supervision; H.L., V.M. performed research; H.L., V.M., C.K., S.Z., C.M.B., J.H.K., S.P., P.P. analyzed data; H.L., V.M., C.A. wrote the paper; S.Z., A.B., N.P., P.A., T.J.M, C.B., G.F.W., and F.J.H. provided insightful comments and contributed in writing, reviewing, and editing.

## Funding

This study was supported by the NIH Grant R37CA239039 (NCI) and the Focused Ultrasound Foundation (AWD 004091).

## Conflicts of Interest

Chetan Bettegowda is a consultant for Haystack Oncology, Bionaut Labs, Privo Technology. Chetan Bettegowda is a co‐founder of OrisDx and Belay Diagnostics.

## Supporting information




**Supporting File**: advs73481‐sup‐0001‐SuppMat.docx.

## Data Availability

All data needed to evaluate the conclusions in the paper are present in the paper and/or the Supplementary Materials. Any additional requests for information can be directed to, and will be fulfilled by, the corresponding authors. Source data are provided with this paper. ML‐CL and FUS data is available at https://github.com/UBBL‐repo/ML‐CL; NP and LB data is available at https://www.synapse.org/Synapse:syn66364497/wiki/, with SynID: syn66364500.
