# Supplementary material for: Data‐Driven Feedback Identifies Focused Ultrasound Exposure Regimens for Improved Nanotheranostic Targeting of the Brain
Source: Adv Sci (Weinh). 2026 Jan 7;13(13):e17834. doi: 10.1002/advs.202517834 (PMC12955872; doi:10.1002/advs.202517834)
Supplement: Supplementary file 1 — Supporting File: advs73481‐sup‐0001‐SuppMat.docx. [file ADVS-13-e17834-s001.docx]

**Supporting Information**

**Data-driven feedback identifies focused ultrasound exposure regimens for improved nanotheranostic targeting of the brain**

*Hohyun Lee,^1,§^ Victor Menezes,^1,§^ Shiqin Zeng,^2^ Chulyong Kim,^1^ Cynthia M. Baseman,^3^ Jae Hyun Kim,^1^ Samhita Padmanabhan,^4^ Pranav Premdas,^5^ Naima Djeddar,^6^ Anton Bryksin,^6^ Nikhil Pandey,^7^ Pavlos Anastasiadis,^7,8,9,10^ Anthony J. Kim,^7,10^ Tobey J. MacDonald,^11^ Chetan Bettegowda,^12^ Graeme F. Woodworth,^7,8,9,10^ Felix J. Herrmann,^2,5,13^ and Costas Arvanitis^1,4*^*

**Affiliations**

^1^ Woodruff School of Mechanical Engineering, Georgia Institute of Technology, Atlanta, Georgia, United States

^2^ School of Computational Science and Engineering, Georgia Institute of Technology, Atlanta, Georgia, United States

^3^ School of Interactive Computing, Georgia Institute of Technology, Atlanta, Georgia, United States

^4^ Coulter Department of Biomedical Engineering, Georgia Institute of Technology and Emory University, Atlanta, Georgia, United States

^5^ School of Electrical and Computer Engineering, Georgia Institute of Technology, Atlanta, Georgia, United States

^6^ Institute of Bioengineering and Bioscience, Georgia Institute of Technology, Atlanta, Georgia, United States

^7^ Department of Neurosurgery, University of Maryland School of Medicine, Baltimore, Maryland, United States

^8^ Brain Tumor Program, Marlene and Stewart Greenebaum Comprehensive Cancer Center, University of Maryland, Baltimore, Maryland, United States

^9^ Department of Diagnostic Radiology and Nuclear Medicine, University of Maryland School of Medicine, Baltimore, Maryland, United States

^10^ Department of Bioengineering, University of Maryland, College Park, Maryland, United States

^11^ Aflac Cancer & Blood Disorders Center, Department of Pediatrics, Emory University School of Medicine, Atlanta, Georgia, United States

^12^ Department of Neurosurgery, John Hopkins University School of Medicine, Baltimore, Maryland, United States

^13^ School of Earth and Atmospheric Sciences, Georgia Institute of Technology, Atlanta, Georgia, United States

^§^**Equally contributing authors**

^*^**Corresponding author:** Costas Arvanitis ([costas.arvanitis@gatech.edu](mailto:costas.arvanitis@gatech.edu))

**Experimental Section and Methods**

1. **Training of different machine learning models with past dataset**

Past acoustic emission (AE) datasets from blood-brain barrier (BBB) opening experiments can be used to train different machine learning (ML) models. Apart from the multilayer perceptron (MLP) model ^[1]^ that we mainly incorporated in the study, we additionally trained support vector machine (SVM) ^[2]^ models in MATLAB (**Figure S1**). All types of models showed similar accuracy, precision, recall (sensitivity), specificity, and f1 score (**Figure S2**). They were also trained in 1) different kernels for SVM – RBF and linear, 2) different loss for MLP – cross entropy (MLP-CE) and mean squared error (MLP-MSE), and 3) different minority sampling methods, such as synthetic minority oversampling technique (SMOTE) and undersampling. Using SMOTE tend to have high precision (false positive vs. true positive), but lower sensitivity (true positive rate). All under-sampled models showed more conservative behavior (i.e., higher false positive) while attaining sensitivity (i.e., true positive rate – correct broadband emission prediction) compared to oversampling technique (**Figure S1 & Figure S2**).


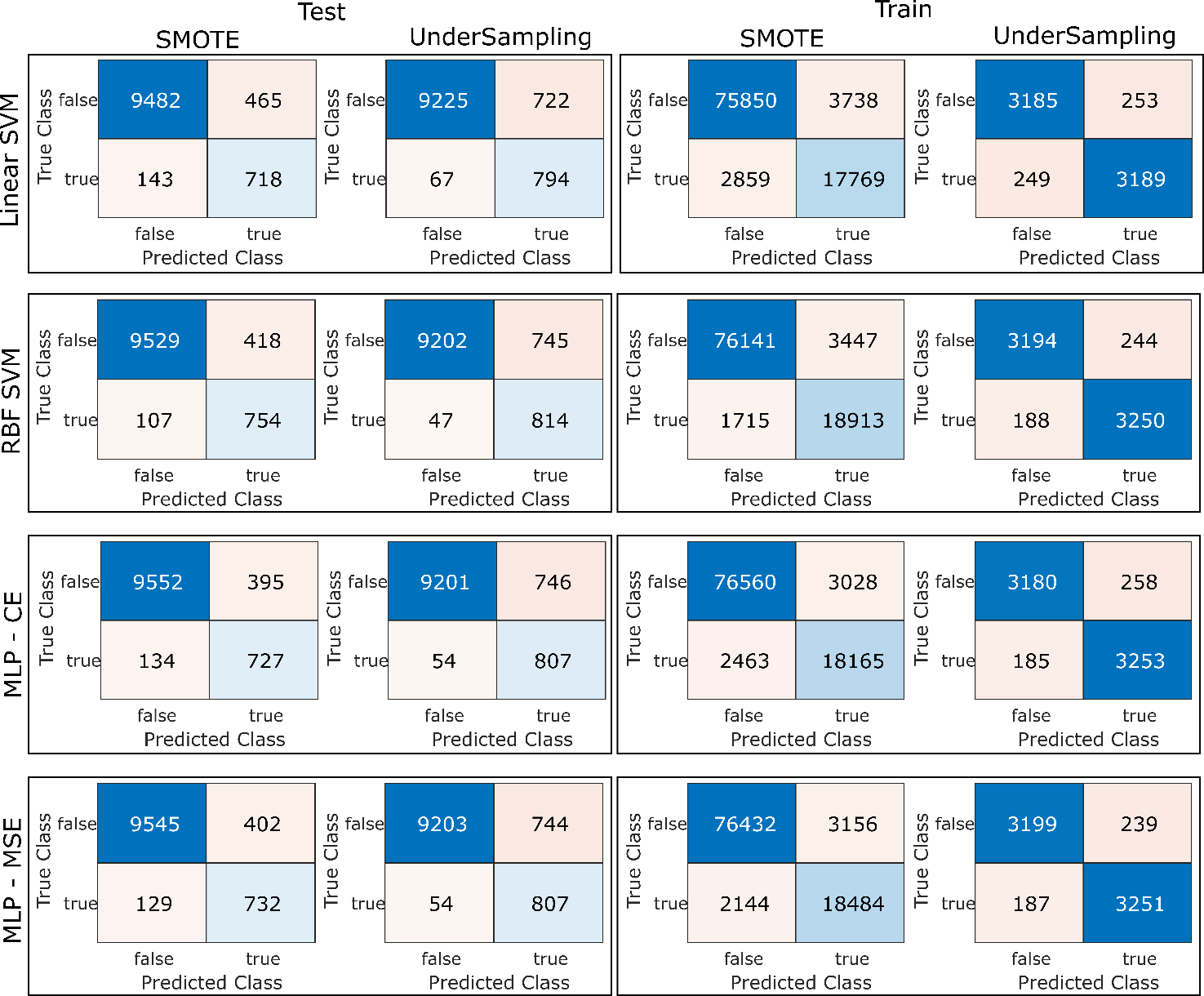


**Figure S1.** Evaluation of different ML algorithms on training dataset: Linear kernel SVM, RBF kernel SVM, MLP with cross-entropy, and MLP with mean squared error (MLP-MSE, the model used for ML-CL). Left column shows performance on the testing dataset, and right column shows performance on the training dataset. For each algorithm, the effect of undersampling or synthetic minority oversampling (SMOTE) was also compared.


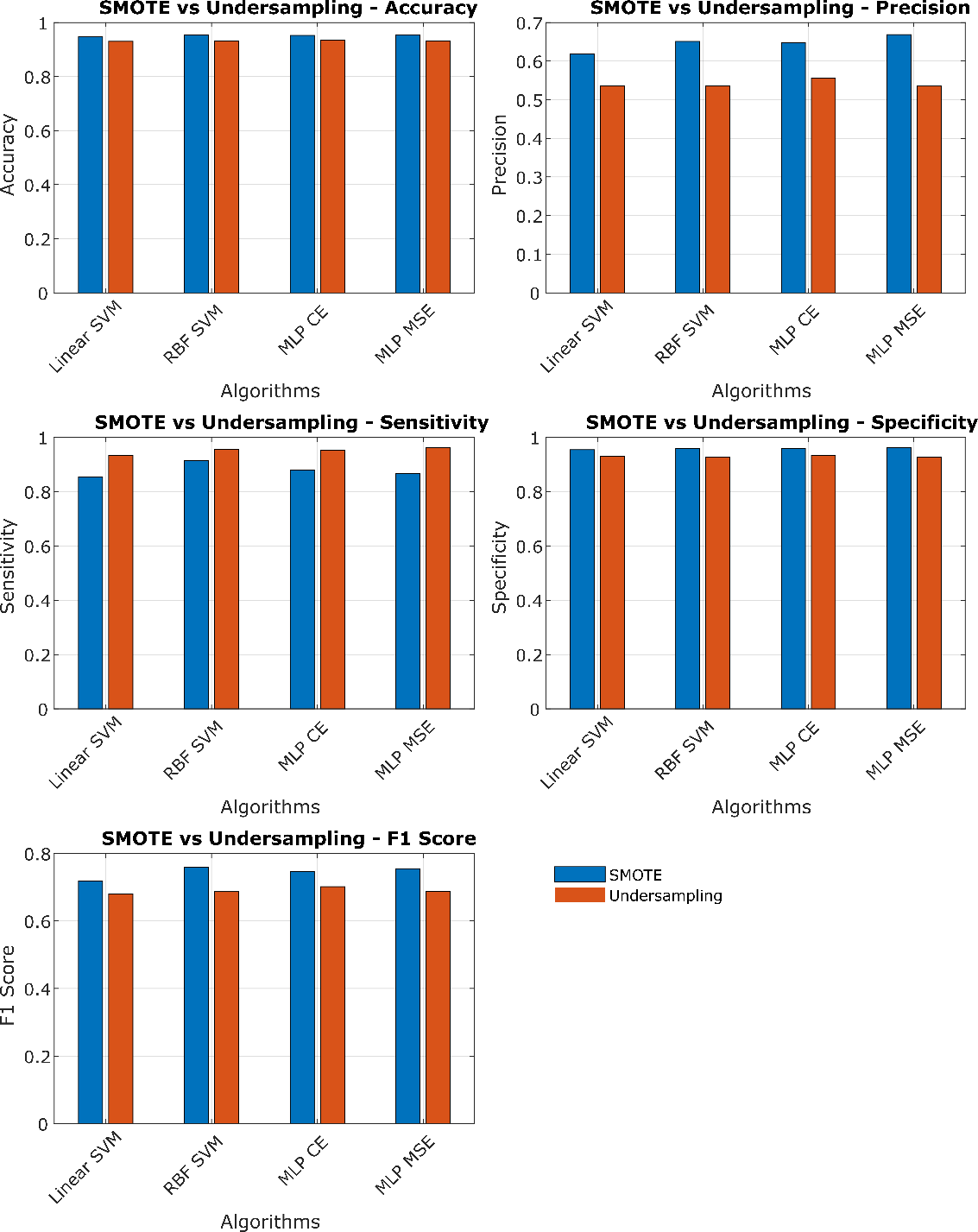


**Figure S2.** Quantification of each ML algorithm's performance. All algorithms showed similar metrics: Accuracy, precision, specificity, sensitivity, and F1 score, given the same minority sampling method. Algorithms trained with SMOTE sampling technique showed tendency of low false positive, but high false negatives.

1. **Training advanced deep learning model (attention mechanism) with past dataset**

Past AE datasets can also be used for training a deep learning model. Inspired by attention mechanisms, which have demonstrated the ability to selectively focus on the most relevant features of input data, leading to state-of-the-art performance across multiple domains, including natural language processing ^[3]^ and computer vision ^[4]^, we extended the MLP concept to context-aware modeling. The proposed model integrates cross-attention to allow patient-specific features (query) to dynamically interact with frequency components (harmonics and ultra-harmonics) from broadband acoustic emissions (key-value pairs). This enables the model to selectively prioritize informative spectral biomarkers while suppressing noise, enhancing predictive accuracy for broadband emission, and ensuring a controlled physiological state. The motivation for applying attention is that traditional MLPs treat all input features equally, whereas attention mechanisms facilitate dynamic feature interactions, allowing the model to adaptively weigh critical signals in relation to the individual characteristics. By leveraging cross-attention, this attention algorithm enables individual non-MBAE features (e.g., MB Kinetics, tumor presence, etc.) to be processed in the context of real-time environmental stimuli, allowing for an adaptive decision-making process to assess MB dynamics is at risk of instability.

The attentive multilayer perceptron (AMP) architecture is illustrated in **Figure S3**. In this framework, individual-specific features first pass through a multilayer perceptron, encoding the information into a latent representation as the query (*Q*), while real-time frequency emission measurements are processed through another MLP, serving as the key (*K*) and value (*V*). The core of the architecture is the scaled dot-product mechanism, which enables interaction between these two feature sets. *Q* is derived from subject-specific features ($f_{i}$), which encode the physiological state of the subject before sonication, $f_{i}$ define the individualized treatment message that the model needs to consider when deciding whether MB dynamics remain controlled or at risk of instability. *K* is from real-time frequency feedback ($f_{e}$), reflecting the acoustic response from the AE measurements during the sonication, which contains harmonic and ultra-harmonic frequency measurements. The role of the *K* is to provide a reference against which the patient-specific information (*Q*) can be compared. The dot product computation of *QK^T^* produces a score matrix where each piece of information interacts with each measurement in the latent space. After normalization via SoftMax, we can get attention scores on how much focus each feature should have on the corresponding real-time feedback. The *V* matrix contains the actual measured frequency emission information, and multiplying the attention score matrix by *V* produces an adaptive and feedback-aware feature representation for further classification. The key advantages of AMP lie in its ability to treat subject-specific and real-time feedback features separately while enabling context-aware interactions in the latent space. The attention mechanism employs three learnable transformation matrices, allowing the model to prioritize relevant frequency components dynamically, suppress noise, and thus enhance feature selection automatically. Besides, the designed architecture ensures that the patient's state is continuously re-evaluated in real time based on AE feedback, improving the accuracy and robustness of broadband emission prediction.


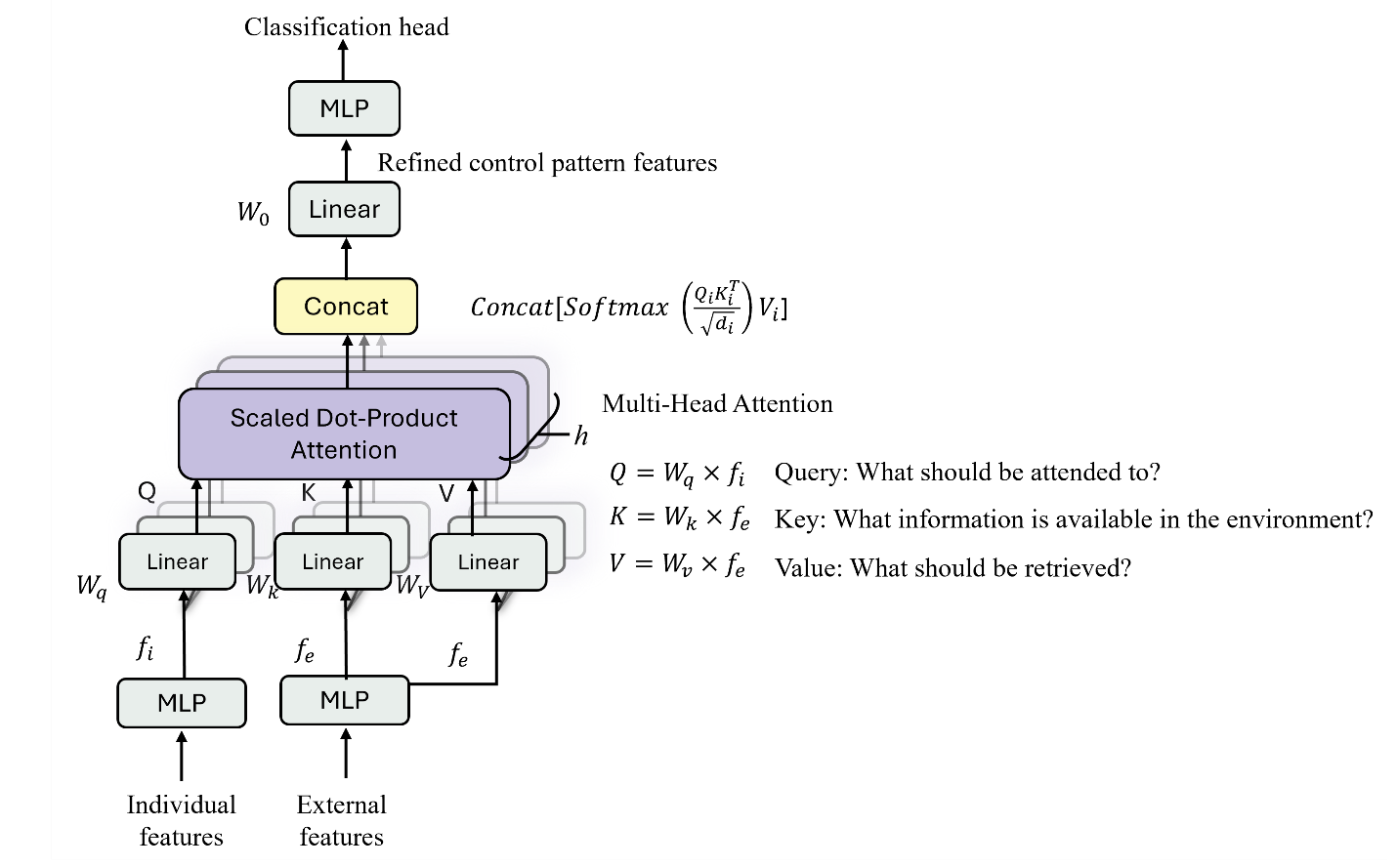


**Figure S3.** Attention multilayer perceptron (AMP) architecture.

1. **Evaluation of attentive multilayer perceptron (AMP)**

The dataset was formed using past AE data that utilized constant pressure sonication from a total of 114 mice. We categorize the input features into two groups: (1) Patient-specific features, which include the target region in the brain (x and y coordinates referenced to the eyes), pulse number, MB kinetics, pressure, and presence or absence of tumor; and (2) Real-time treatment feedback, which consists of broadband emission extracted from AE signals. These signals were selected to be the current (t_n_) 2~8^th^ (2~8f_0_) harmonic emission levels 1~7^th^ ultra-harmonic (1.5~7.5f_0_) emission levels. The labels for supervised machine learning classification were binary indicators of broadband emissions exceeding 6 dB above the baseline at the subsequent sonication (t_n+1_). The training dataset was structured as an $N\times[D_{1}, D_{2}]$ matrix, where $N$ presents the total number of AE samples ($N=54,040$), $D_{1}$corresponds to the patient-specific feature dimension ($D_{1}=6$), and $D_{2}$ represents the real-time frequency-driven treatment feedback measurements ($D_{2}=[7, 7]$). The corresponding labels were stored as an $N\times1$ vector. Since broadband emissions above 6 dB were rare, accounting for only 8% of the total dataset (4,299 out of 54,040), we maintained this ratio when splitting the data into training (80%) and test (20%) sets to reflect the real data distribution. To address this class imbalance during training, we employed weighted cross-entropy loss, assigning a weight of ($\frac{54040}{4299}\times\varepsilon$) to broadband emissions exceeding 6 dB classes (in our case, we assign $\varepsilon=2$). Additionally, we applied K= 10-fold cross-validation in the training dataset to determine the optimal hyperparameters and select the best performance model. To mitigate overfitting, dropout, L2 regularization, and layer normalization were incorporated into the training process.

We found that compared to MLP, AMP had a decreased false positive rate (**Figure S4A**). Moreover, SHAP analysis of the AMP model (**Figure S4B**) provided similar results to our MLP SHAP analysis (**Figure 1D**).


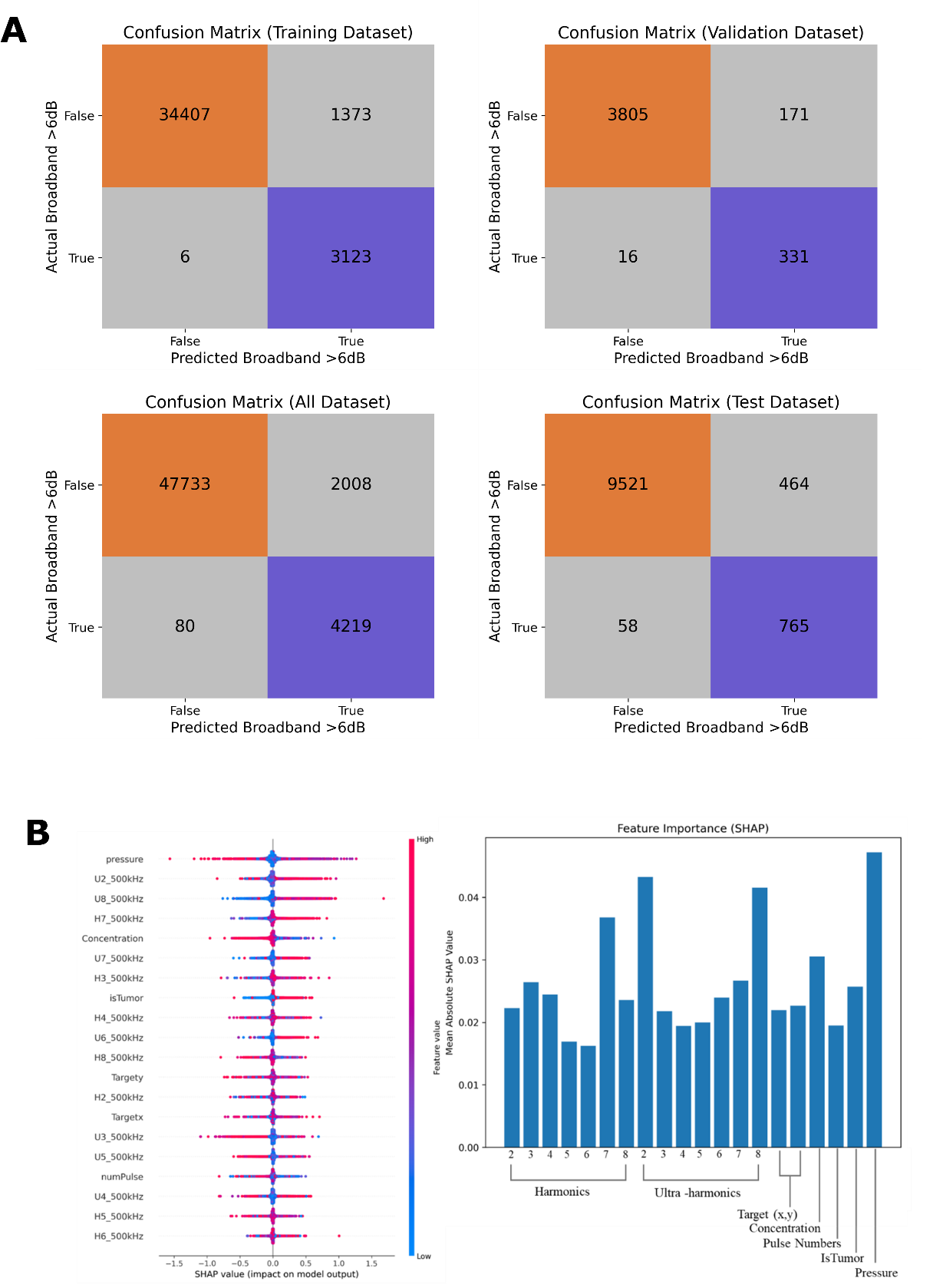


**Figure S4.** A) Confusion matrix of AMP in training, validation, testing, and overall dataset. Compared to MLP only, AMP showed general improvement in lower false positives. B) Shapley additive analysis (SHAP) onto AMP. MLP and AMP had similar importance in top features.

1. **Correlation between MB kinetics and broadband emission strength obtained from the training dataset**

We analyzed the training dataset further to find the relationship between broadband emission strength and MB kinetics. We correlated the MB kinetics (transient decay in harmonic emission, normalized to maximum harmonic emission level) to broadband emission. Our analysis showed that stronger broadband emission (>15 dB) has maximum likelihood at higher MB kinetics (i.e., right after bolus injection of MBs) (**Figure S5**). This may suggest the inherent risk of using constant pressure sonication; at the least, constant pressure sonication should be started after a few seconds of MB administration.


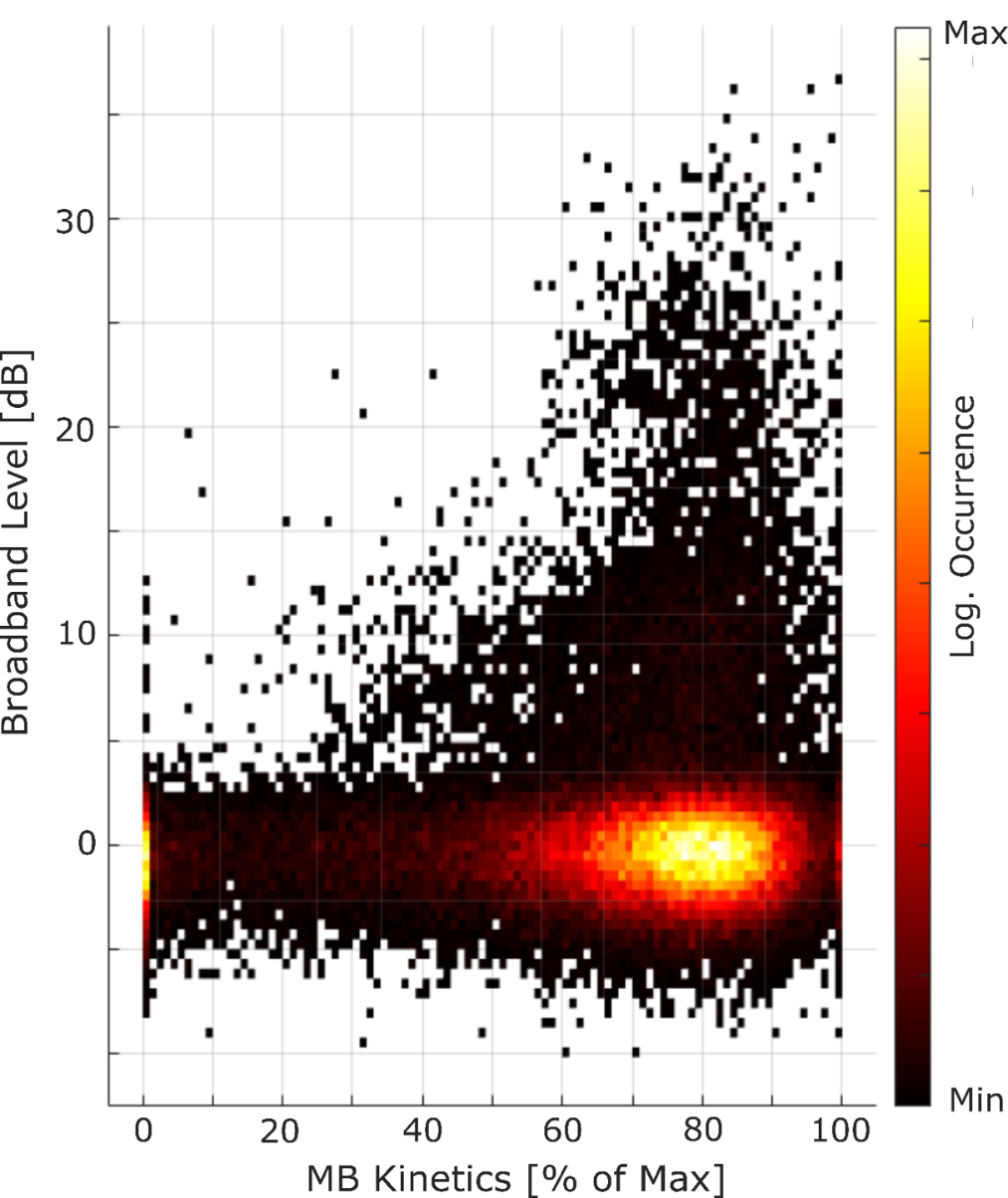


**Figure S5.** 2D histogram of Broadband emission level as a function of MB kinetics. Colorbar indicates the logarithm of the number of occurrences.

1. **Application of machine learning-assisted closed-loop controller (ML-CL) onto healthy mouse with 32 dB target level**

To evaluate the operation of the ML-assisted closed-loop controller (ML-CL), we first performed MB-FUS mediated BBB opening in healthy mice by targeting three regions in each brain hemisphere using the USgFUS system that we used to collect the training data. We selected a target level of 32 dB at 7^th^ harmonic emission using the cavitation threshold curve identified from our training dataset (**Figure S6A**). We chose this target level because it was at the highest level within the linear regime of the curve. With a 32 dB target level, we found that the average and maximum pressure decisions (P_Avg_ and P_Max_, as reported as peak negative pressures) of ML-CL were 0.17 MPa and 0.23 MPa, respectively (**Figure S6B**). Along with the pressure corresponding to 32 dB in the cavitation threshold model (P_Model_ = 0.14 MPa, **Figure S6A**), these pressures provided the necessary exposure conditions for implementing constant pressure open-loop controllers (OL) to compare and benchmark the ML-CL controller’s performance. This was performed by comparing the resulting AE, total broadband emission events, and BBB opening strength quantified using dynamic contrast-enhanced MRI (DCE-MRI) in healthy mice (**Figure S6C-D**). Note that every controller incorporated MB kinetics tracking pulse (a small constant pressure pulse to monitor MB kinetics) to begin their operation upon detection of MB arrival to the brain (**Figure S6B**).

We found that ML-CL achieved 31.6 ± 0.6 dB 7^th^ harmonic emission level with significantly lower AE fluctuation (i.e., more stability and ability to achieve and maintain prescribed level) compared to P_Model_ (24.4 ± 3.5 dB) and P_Avg_ (27.5 ± 4.7 dB) OL controllers (**Figure S6E**), because of the feedback algorithm. In line with the AE levels, we observed a similar trend in K_trans_ values derived from DCE-MRI across different controllers (**Figure S6F-G**). Although P_Max_ (31.7 ± 0.7 dB) showed comparable AE performance and higher K_trans_ compared to ML-CL, it also exhibited 3 instances of broadband emission during sonication (**Figure S6H**), which indicates that safety was compromised. On the other hand, we observed that ML-CL controller had predicted and responded to 0.2% (3/1820) instances of potential broadband emission events (**Figure S6I**) during its real-time operation. Post-sonications analysis further indicated that if it was integrated into the OL controller, the MLP model could have predicted all broadband emission events (3/4680) during the OL controller’s operation (**Figure S6I**).


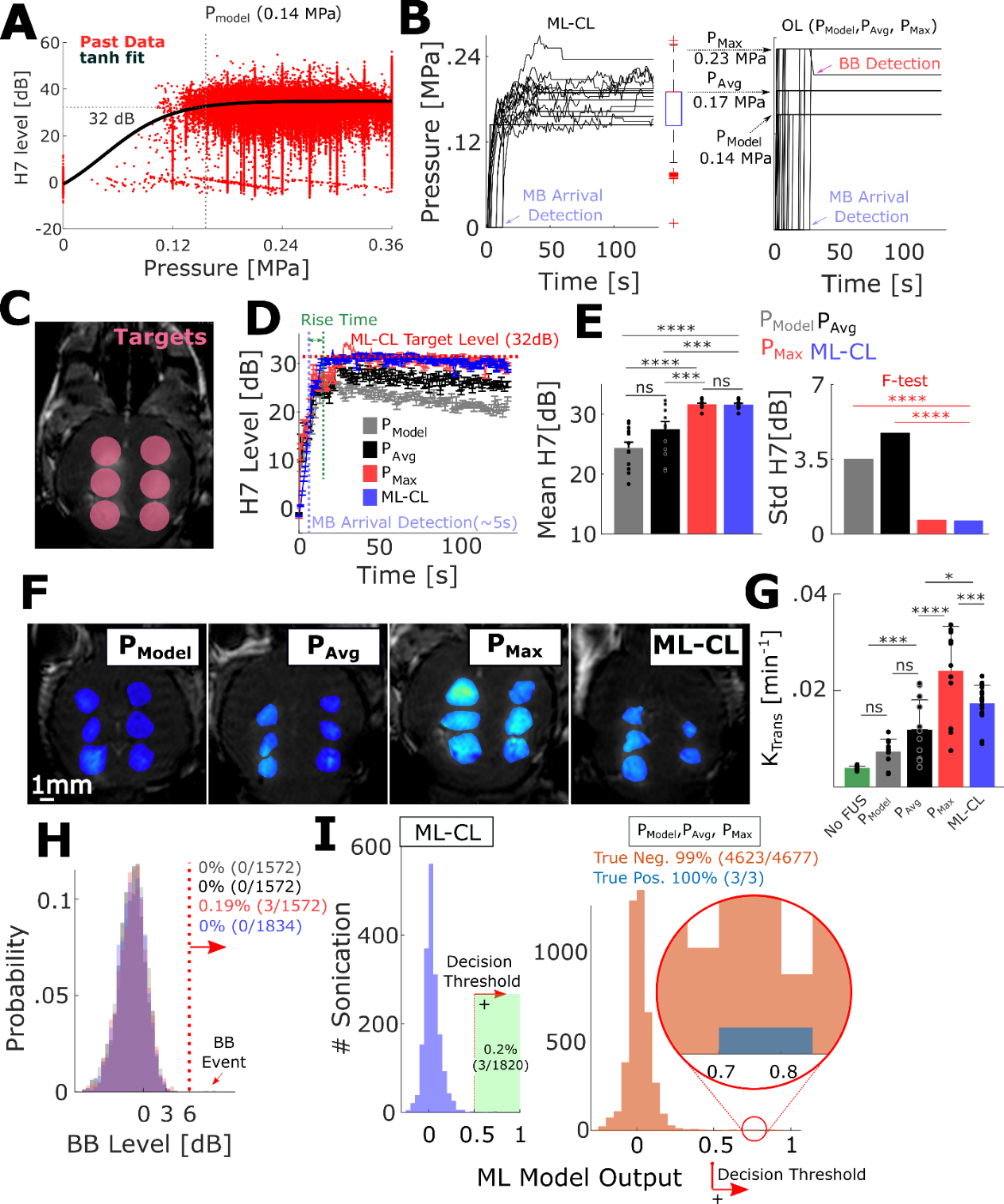


**Figure S6.** Performance assessment of ML-CL in BBB opening with 32 dB target level. **A)** Cavitation threshold model from the training dataset. 7^th^ harmonic AE as a function of pressure. The model can be used to determine an adequate target level for the controller, and the P_Model_ is determined here as 0.14 MPa. **B)** ML-CL pressure decisions for 32 dB target level (left). The average and maximum pressures used by ML-CL are used to determine P_Max_ (0.17 MPa) and P_Avg_ (0.23 MPa) for OL controllers (right). A decrease in pressure in OL indicates a reaction to broadband emission event (> 6dB). **C)** Representative MRI image for 32 dB target level sonication targets. 3 targets in each brain hemisphere (total 6 targets per mouse) were treated. **D)** 7^th^ harmonic emissions during sonication for each controller. n = 2 animals (total 12 targets) per group. **E)** Mean (left) and standard deviation (right) of 7^th^ harmonic emission for ML-CL. A variance test (f-test) was performed to compare standard deviation (right). **F)** MRI T1 images using the controller at 32 dB or equivalent OL pressure. **G)** Quantification of K_trans_ values through DCE-MRI. **H)** Histogram for broadband emission levels during sonication. Broadband emissions higher than 6 dB were considered a broadband emission event, whose probability for each controller is highlighted next to the dotted line. **I)** Histogram for MLP decision during ML-CL sonication (left). A model output of 0.5 or greater was considered a positive prediction. For this target level (32 dB), the model predicted 0.2% (3/1820) of sonication. Application of MLP onto OL algorithms (right). Orange indicates true negative accuracy (predicting no broadband emission) and blue indicates true positive accuracy (predicting broadband emission). *p<0.05, **p<0.01, ***p<0.001, and ****p<0.0001. ns = not significant. Statistical analyses were performed through One-way ANOVA and Bonferroni correction.

1. **Post-sonication application of MLP onto 36 dB OL controllers**

We applied MLP to predict broadband emissions on the acquired AE dataset after OL (P_Max_ and P_Avg_, 0.33 and 0.25 MPa, respectively) sonication. MLP was able to predict 51% of the existing broadband emission events that were observed during sonication (**Figure S7**).


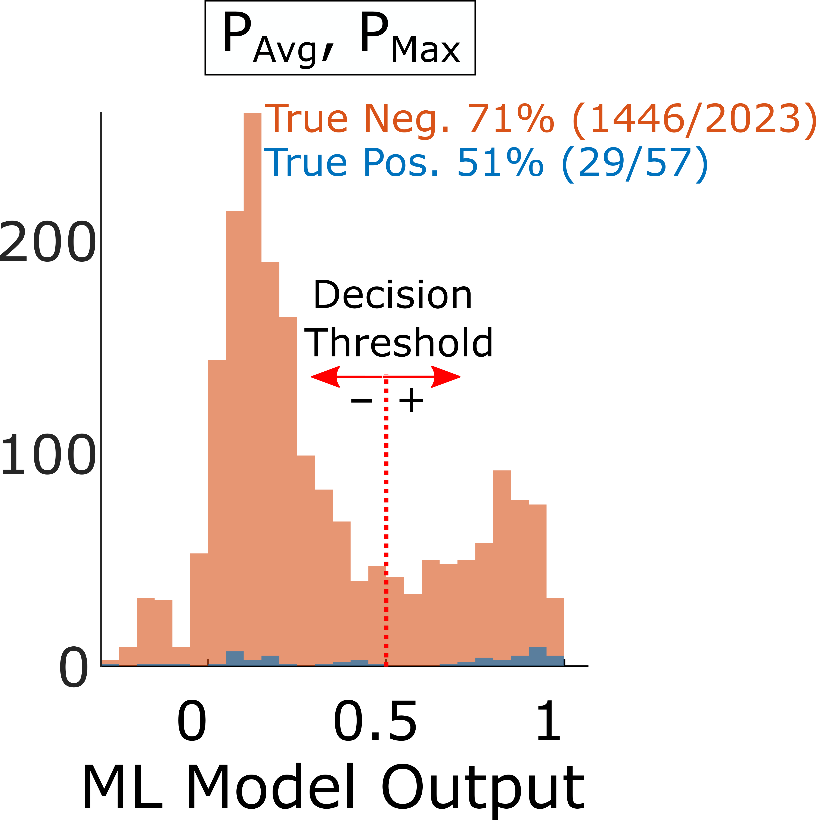


**Figure S7.** Post-sonication application of MLP onto OL (P_Max_ and P_Avg_, 0.33 and 0.25 MPa, respectively) sonication. MLP was able to predict 51% of the existing broadband emission events that were observed during sonication. Orange indicates true negative accuracy (predicting no broadband emission), and blue indicates true positive accuracy (predicting broadband emission).

1. **Broadband emission and real-time pressure**

For more detailed information of **Figure 4J**, pressure vs. broadband level throughout all sonication AE datasets (5240 in total) was included in this Supplementary Information with a more detailed figure of **Figure 4J** with individual data points (**Figure S8**). The majority of data (due to scarcity of broadband emissions) were around noise level (less than 4 dB). Thus, the broadband emissions for each controller at the noise level were downsampled with a 1% rate to obtain the fit of the hypertangent curve. There was 34% increase in the inflection point (i.e., curve shift), whose pressures were used to determine the upperbound of the therapeutic window.


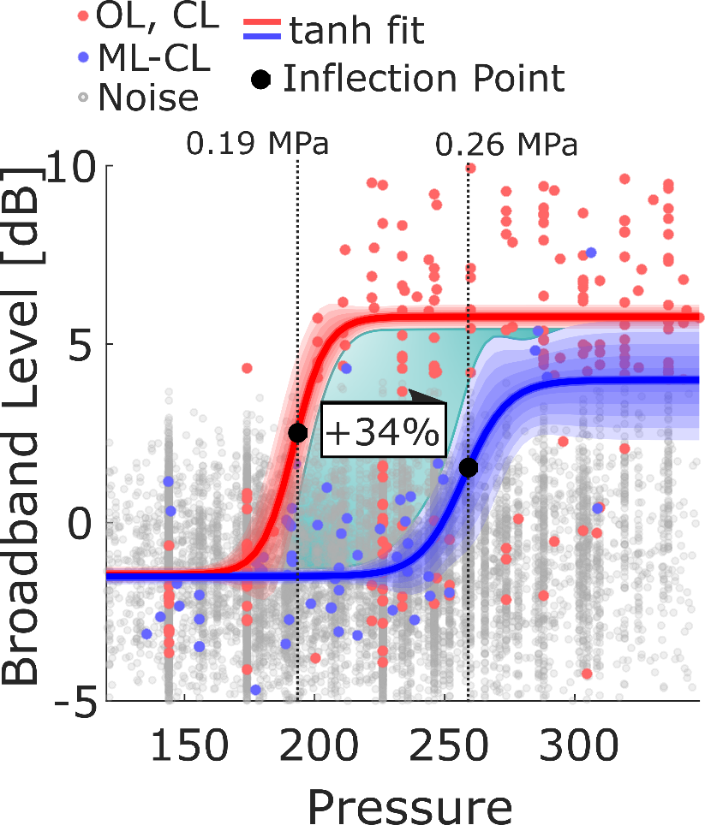


**Figure S8.** Pressure vs. broadband level throughout all sonication AE dataset (5240 in total). A more detailed figure of **Figure 4J** with individual data points. The majority of data (due to scarcity of broadband emissions) were around noise level (less than 4 dB). Thus, the broadband emissions for each controller at the noise level were downsampled with 1% rate to obtain the curve. There was 34% increase in the inflection point (i.e., curve shift), whose pressures were used to determine the upperbound of the therapeutic window.

1. **Performance of ML-CL in safety**

In assessing ML-CL’s safety with 36 dB, we assessed whether there were any false negatives (no broadband but tissue damage) after sonication. We found that CL contained 2 out of 8 targets where no broadband was observed but had hemorrhage (**Figure S9**). This case was also found at P_Avg_ sonication.


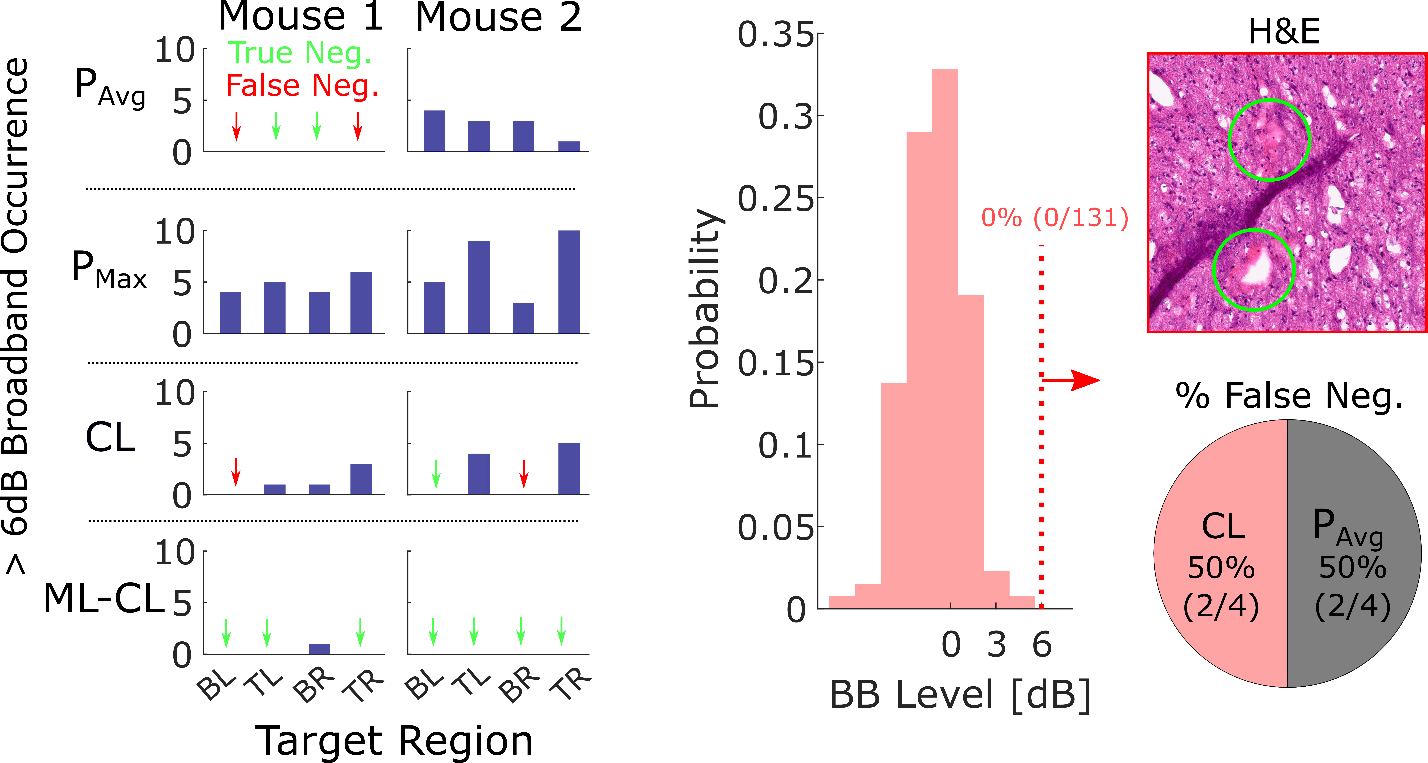


**Figure S9.** Presence of broadband emission at BL (bottom-left), TL (top-left), BR (bottom-right), and TR (top-right) target locations. Green arrows indicate true negative (no broadband and no damage), and red arrows indicate false negative (no broadband and damage). Also, a Histogram of broadband emission for BL target in CL algorithm where no broadband was observed but petechiae was observed. Bottom right: pie chart for each controller’s stake in false-negative events.

1. **Performance of ML-CL in nanoparticle delivery**

In applying ML-CL onto different sizes of nanoparticle delivery, we applied 32 dB and 36 dB target level ML-CL onto the left and right side of healthy brain hemispheres, respectively. The pressure used by ML-CL for each the target levels was notebly different (**Figure S10A**), which resulted in significantly different (p=0.009) 7^th^ harmonic emissions (**Figure S10B**). Broadband emission event (>6dB) was absent except for one event at 120 nm sonication with 36 dB ML-CL (**Figure S10C**).


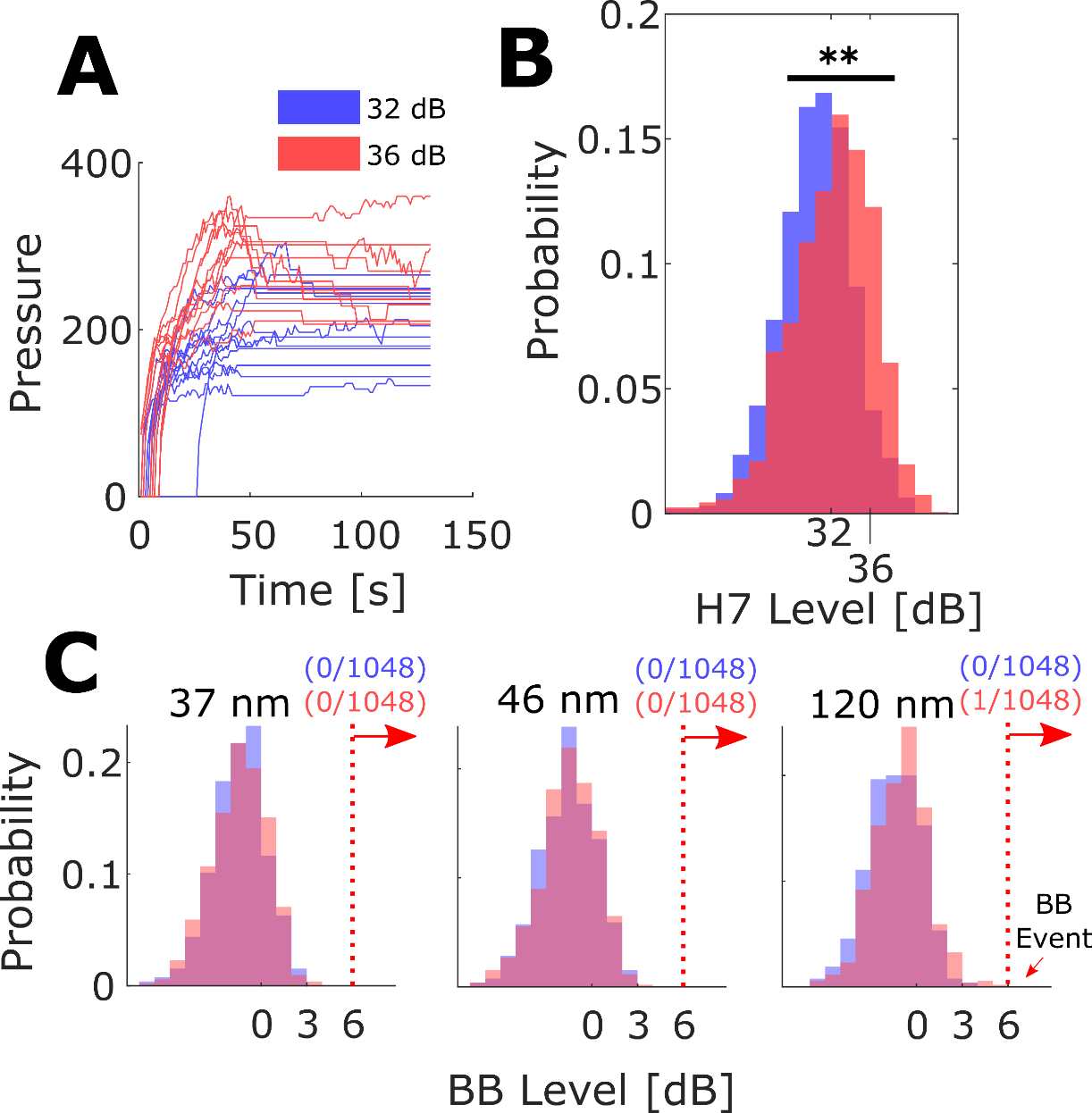


**Figure S10. A)** Pressures used by ML-CL at each (32 and 36 db) target level. **B)** Histogram for 7^th^ harmonic emission during ML-CL sonication. **C)** Histogram for broadband emission during ML-CL sonication. One event was present at 120 nm cohort during 36 dB target level operation. All red colors indicate 36 dB target level, and blue colors indicate 32 dB target level.

1. **Liquid biopsy (LB) with multiple timepoint collection in mice**

For mice, blood samples (180µL/each) were collected retro-orbitally 5 minutes before and 15min after sonication at the treatment midpoint (for both FUS and control groups) and 2-hours-post-treatment (terminal – for ML-CL group) using EDTA-coated capillary tubes attached to a non-coated 1.5mL microcentrifuge tube. All samples were allowed to coagulate in ice for 10 minutes prior to 1,000g centrifugation for 20 minutes. Serum was allocated for protein quantification (20µL – all animals) and ctDNA (80µL for mice and 500 µL for rats) purification/quantification.

We found that the protein concentration in the blood increased by 3.1-fold immediately after sonication, as compared to pre-sonication (p<0.05) for 36 dB ML-CL (**Figure S11**), indicating a burst release of GLuc protein from the tumors to the circulation following ML-CL sonication. Although these levels persisted even after 2 hours, a declining trend was evident (**Figure S11**). In contrast to protein, the GLuc gene analysis showed that, on average, the levels in the number of positive droplets in the circulation with and without MB-FUS were very low and inconsistent.


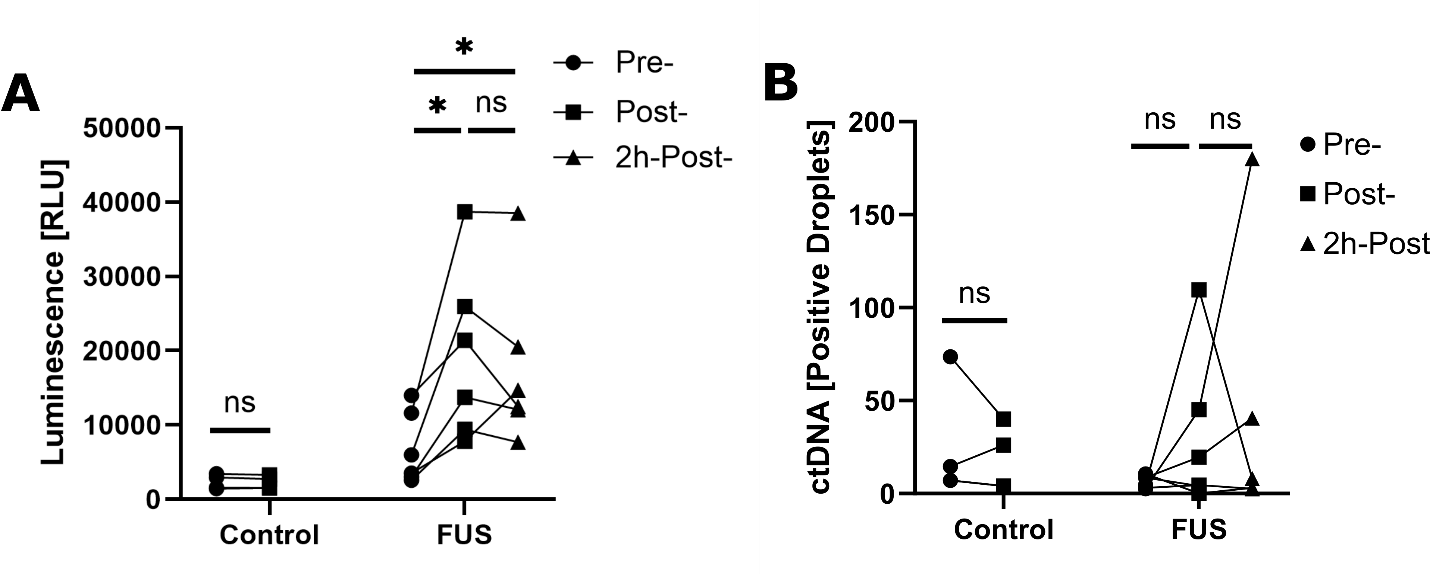


**Figure S11.** Protein and ctDNA quantification at multiple timepoint. Control group includes pre- and post-treatment samples, while FUS group includes pre-, post-, and 2h-post **A)** Protein quantification with luminescent signal acquired (see methods for details of quantification). **B)** ctDNA quantification (positive dPCR droplets).

1. **BBB reversibility after sonication with ML-CL**

To investigate the reversibility of BBB opening using ML-CL and BBB closure, we sonicated healthy rats (n = 4) with 4 targets – 2 targets on left/right hemisphere, with constant pressure (P_Avg_, 0.29 MPa) and ML-CL, respectively. We then assessed the reversibility of BBB opening by monitoring DCE-MR-images (K_trans_) at 15 minutes, 6 hours, and 24 hours post-sonication. At each of time points, we initially performed MR-T1 imaging without contrast agent to confirm their clearance followed by DCE-MRI and another MR-T1 acquisition, both with contrast agents. Our data suggests that BBB opening returns to approximately baseline level (un-sonicated regions) after 6 hours for both sonication protocols. While ML-CL, evidenced by K_trans_ values, showed a significantly stronger BBB opening compared to P_Avg_ and showed a slower trend of BBB reversibility, our data indicated that by 24 hours, BBB reversibility of both sonication protocol closely matched (**Figure S12**). This corroborates previous studies on this matter ^[5]^, where stronger BBB openings – achieved by ML-CL in our work – are correlated with longer BBB reversals.

**
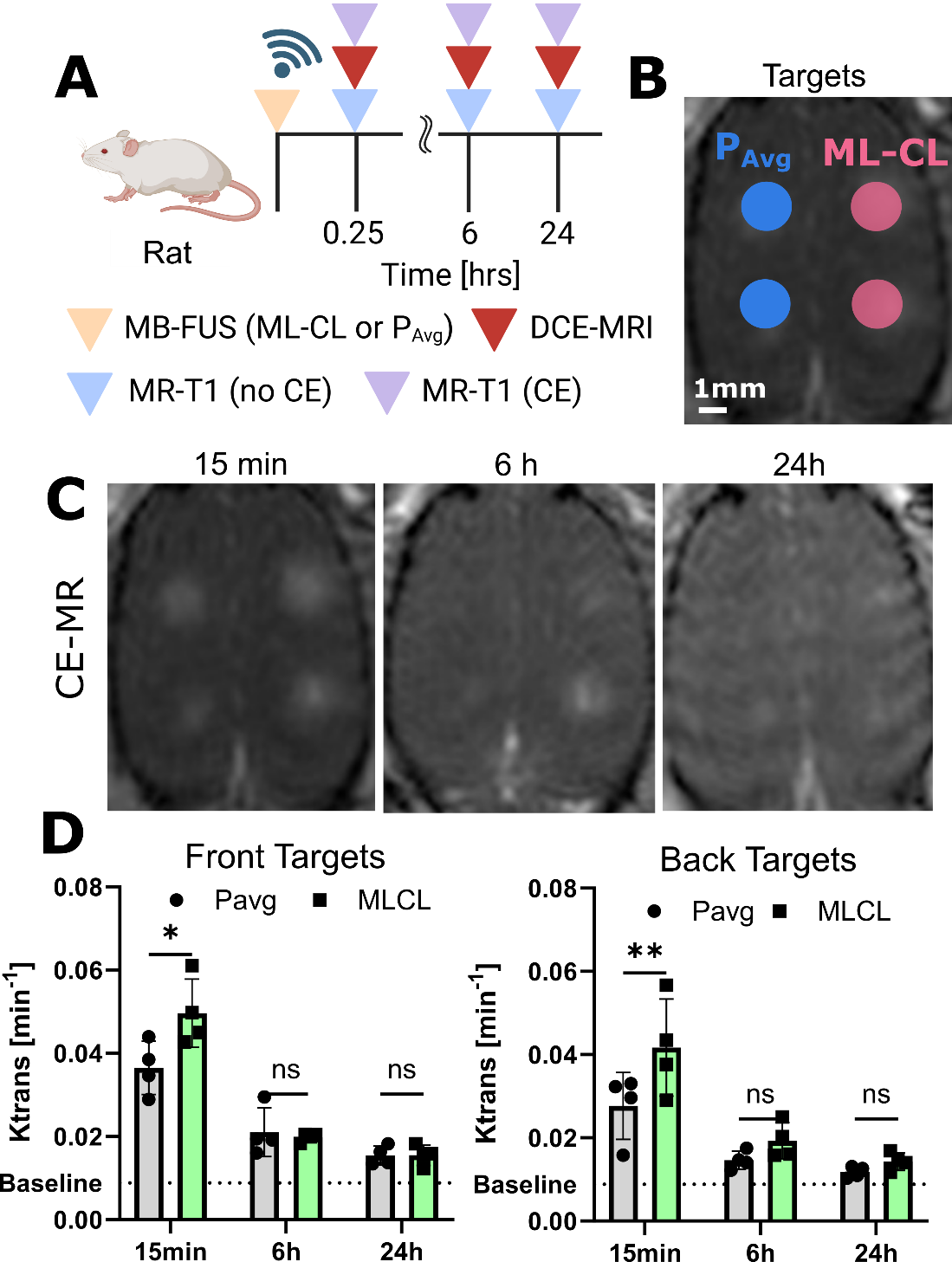
**

**Figure S12.** Investigation on BBB reversibility on healthy rats. **A)** Experiment protocol. After sonication with either ML-CL or P_Avg_, we acquired pre-contrast-enhanced MR-T1 followed by contrast-enhanced DCE-MRI and MR-T1 to track BBB reversibility. **B)** Sonication targets. **C)** Representative CE-MR-T1 images at each time points (15 min, 6 hours, and 24 hours). **D)** K_trans_ analysis at each time points.

1. **Long-term (48 hours) safety investigation**

To investigate longer term safety of ML-CL, we sacrificed the rats in BBB closure cohort (**Figure S12**) 48 hours after sonication (See Methods section for detailed brain tissue processing and immunofluorescence/immunohistochemistry staining and imaging). Both P_Avg_ and ML-CL sonications had less than 0.1% (3/2096 and 2/2096, respectively) broadband emission events. To evaluate potential brain injuries across focal region, we obtained 3 slices of the brains at different depths: one at the intended focal plane, and two additional slices at 1 mm and 2 mm above the focus. We then stained the slides with Gfap, Iba1, and Hematoxylin-eosin (**Figure S13A**). Overall, no significant differences in Iba1 or Gfap expressions were observed between ML-CL and P_Avg_ (**Figure S13B**), Moreover, localized increase signal intensities at the focal region (**Figure S13C**) suggest that any potential adverse effects are spatially confined, confirming that our safety assessments effectively capture the extent of MB-FUS-induced tissue responses.

**
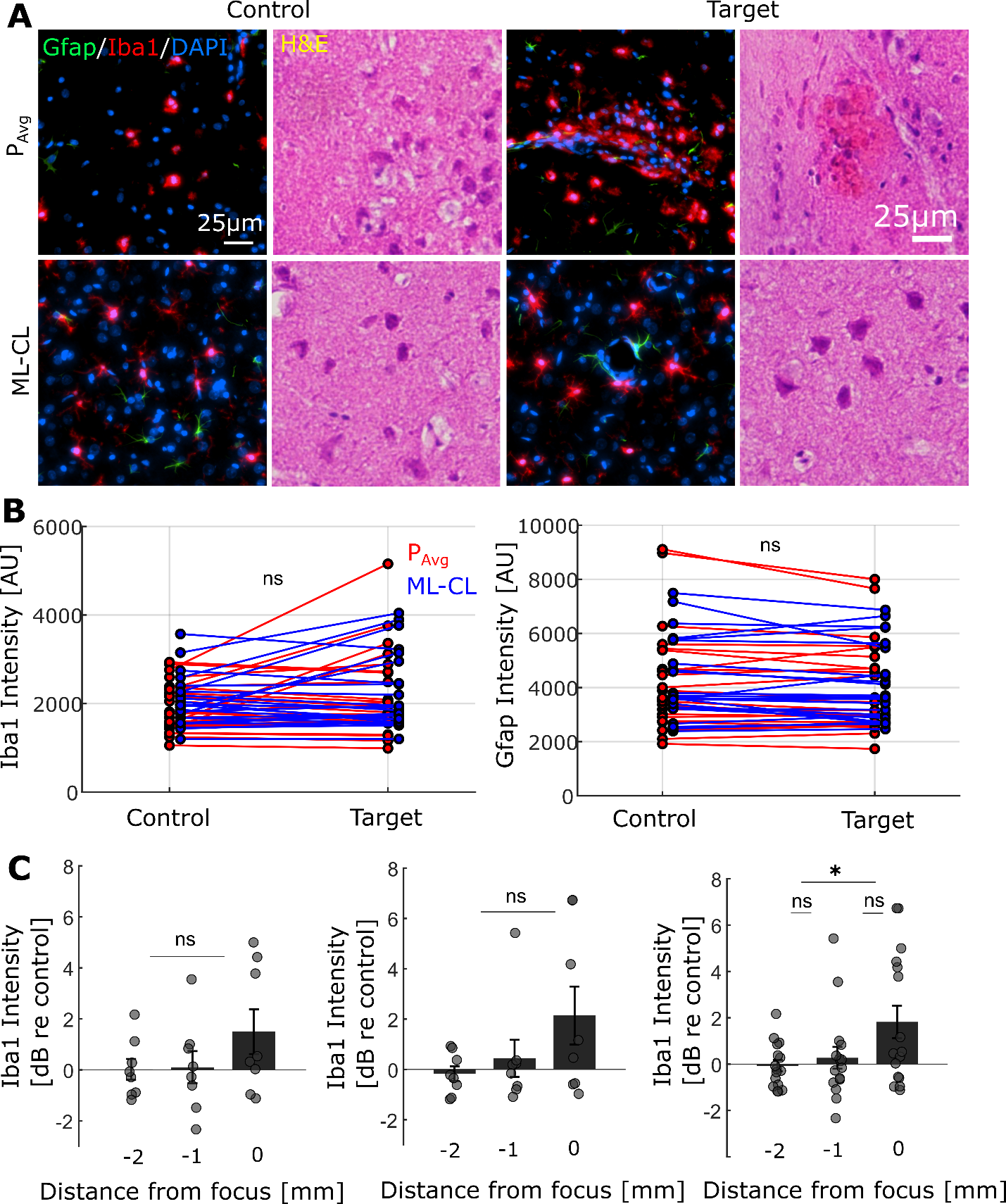
**

**Figure S13.** Investigation on long term safety (48 hours) on healthy rats. **A)** Representative Iba1, Gfap, and H&E staining 48 hours after sonication. Control indicates non-sonicated brain sites. **B)** Quantification Iba1 and Gfap at different targets and depths (n = 24 data per group: 4 animals, 2 targets, 3 slices per group). Pairwise plot connecting non-sonicated site with the treatment site. **C)** Tissue depth (distance from focus) versus Iba1 intensity, where dB re control represents quantification in decibel scale with respect to control (non-sonicated site). Left: P_Avg_, Middle: ML-CL, and Right: P_Avg_ and ML-CL. Error bars indicate SEM. *p<0.05, ns = not significant. One-way ANOVA with Bonferroni multiple comparison correction was used.

1. **Liquid biopsy (LB) rat strain impact of biomarker baseline concentration**

Biomarker quantification in different rat strains (immunocompetent and immunocompromised) indicated that the protein concentration baseline is elevated for immunocompetent rats (**Figure S14**), which is probably due to the low clearance of molecules in circulation^[6,7]^ and retention of biomarkers. The saturation of biomarker concentration does not represent the realistic application of this technique and may diminish the impact of FUS on biomarker release.


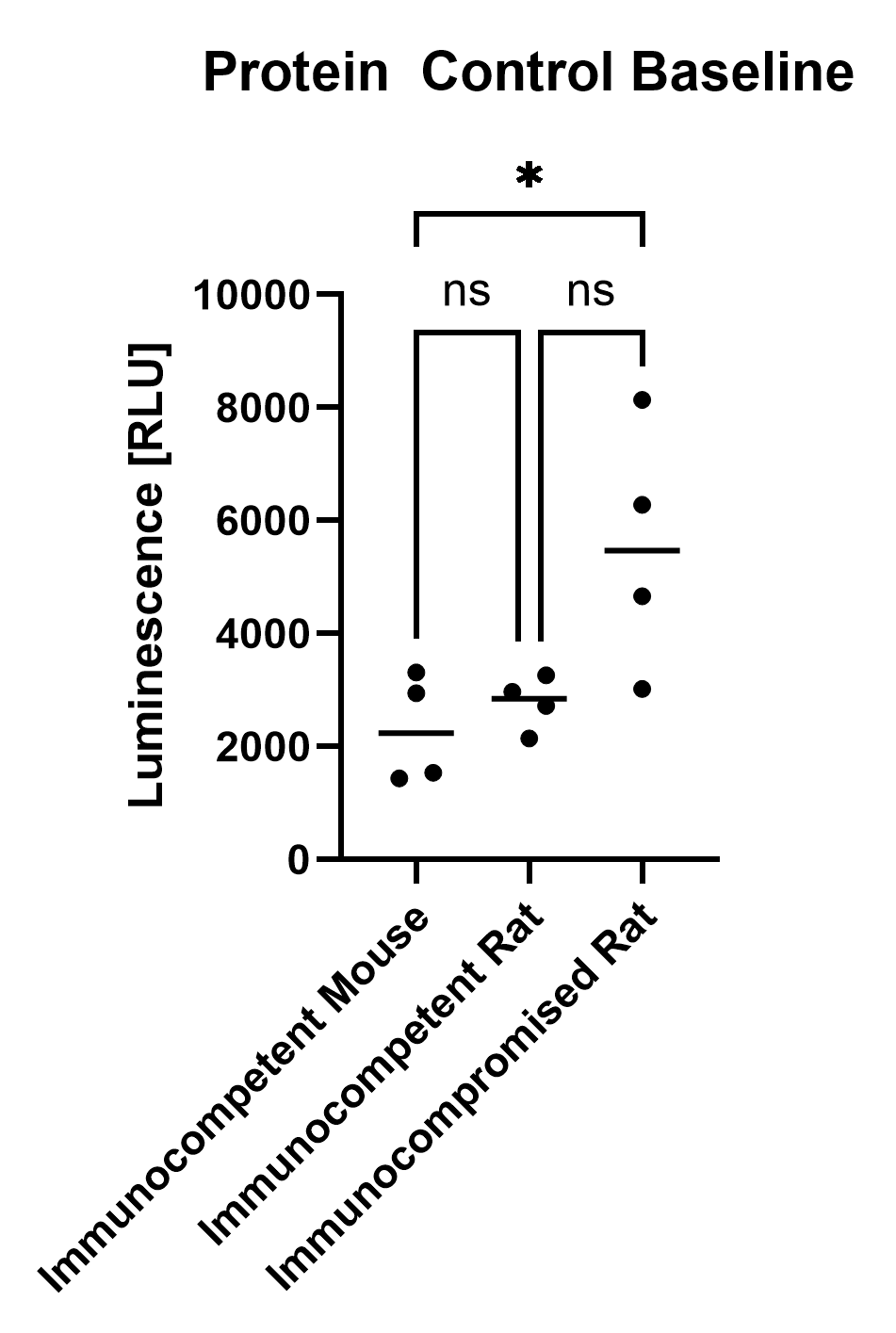


**Figure S14.** Comparison of protein concentration baseline (pre-FUS sonication) for mouse, immunocompromised, and immunocompetent rats.

**References**

1. D. E. Rumelhart, G. E. Hinton, R. J. Williams, *Nature* **1986**, *323*, 533.
2. C. J. C. Burges, *Data Mining and Knowledge Discovery* **1998**, *2*, 121.
3. A. Vaswani, N. Shazeer, N. Parmar, J. Uszkoreit, L. Jones, A. N. Gomez, Ł. ukasz Kaiser, I. Polosukhin, in *Advances in Neural Information Processing Systems*, Vol. 30, Curran Associates, Inc. **2017**.
4. A. Dosovitskiy, L. Beyer, A. Kolesnikov, D. Weissenborn, X. Zhai, T. Unterthiner, M. Dehghani, M. Minderer, G. Heigold, S. Gelly, J. Uszkoreit, N. Houlsby, *An Image is Worth 16x16 Words: Transformers for Image Recognition at Scale*, arXiv **2021**.
5. G. Samiotaki, E. E. Konofagou, *IEEE Transactions on Ultrasonics, Ferroelectrics, and Frequency Control* **2013**, *60*, 2257.
6. C. Odaka, T. Mizuochi, *Cell Death and Differentiation*.
7. Q. Liu, S. Zhou, C. Fan, W. Huang, Q. Li, S. Liu, X. Wu, B. Li, Y. Wang, *Sci Rep* **2017**, *7*, 3597.
